# Isolation, sequencing, and expression analysis of 30 AP2/ERF transcription factors in apple

**DOI:** 10.7717/peerj.8391

**Published:** 2020-01-17

**Authors:** Huifeng Li, Qinglong Dong, Qiang Zhao, Song Shi, Kun Ran

**Affiliations:** 1 Shandong Institute of Pomology, Tai’an, China; 2 College of Horticulture, Northwest A and F University, Yangling, China; 3 College of Horticulture, Qingdao Agricultural University, Qingdao, China; 4 Nanjing Agricultural University, Nanjing, China

**Keywords:** Sequencing, Expression analysis, Apple, AP2/ERF transcription factors, Abiotic stress, Biotic stress

## Abstract

**Background:**

AP2/ERF transcription factors are involved in the regulation of plant growth, development, and stress responses. Our research objective was to characterize novel apple (*Malus* × *domestica* Borkh.) genes encoding AP2/ERF transcription factors involved in regulation of plant growth, development, and stress response. The transcriptional level of apple *AP2/ERF* genes in different tissues and under various biotic and abiotic stress was determined to provide valuable insights into the function of AP2/ERF transcription factors in apple.

**Methods:**

Thirty full-length cDNA sequences of apple *AP2/ERF* genes were isolated from ‘Zihong Fuji’ apple (*Malus* × *domestica* cv. Zihong Fuji) via homologous comparison and RT-PCR confirmation, and the obtained cDNA sequences and the deduced amino acid sequences were analyzed with bioinformatics methods. Expression levels of apple *AP2/ERF* genes were detected in 16 different tissues using a known array. Expression patterns of apple *AP2/ERF* genes were detected in response to *Alternaria alternata* apple pathotype (AAAP) infection using RNA-seq with existing data, and the expression of apple *AP2/ERF* genes was analyzed under NaCl and mannitol treatments using qRT-PCR.

**Results:**

The sequencing results produced 30 cDNAs (designated as *MdERF3-8*, *MdERF11*, *MdERF16-19*, *MdERF22-28*, *MdERF31-35*, *MdERF39*, *MdAP2D60*, *MdAP2D62-65*, and *MdRAV2*). Phylogenetic analysis revealed that MdERF11/16, MdERF33/35, MdERF34/39, and MdERF18/23 belonged to groups A-2, A-4, A-5, and A-6 of the DREB subfamily, respectively; MdERF31, MdERF19, MdERF4/25/28/32, MdERF24, MdERF5/6/27, and MdERF3/7/8/17/22/26 belonged to groups B-1, B-2, B-3, B-4, B-5, and B-6 of the ERF subfamily, respectively; MdAP2D60 and MdAP2D62/63/64/65 belonged to the AP2 subfamily; and MdRAV2 belonged to the RAV subfamily. Array results indicated that 30 apple *AP2/ERF* genes were expressed in all examined tissues to different degrees. RNA-seq results using previously reported data showed that many members of the apple ERF and DREB subfamilies were induced by *Alternaria alternate* apple pathotype (AAAP) infection. Under salt treatment, many members in the apple ERF and DREB subfamilies were transcriptionally up or down-regulated. Under mannitol treatment, many members of the apple ERF, DREB, and AP2 subfamilies were induced at the transcriptional level. Taken together, the results indicated that the cloned apple *AP2/ERF* genes were expressed in all examined tissues. These genes were up-regulated or down-regulated in response to AAAP infection and to salt or mannitol treatment, which suggested they may be involved in regulating growth, development, and stress response in apple.

## Introduction

AP2/ERF is one of the large transcription factor families in plants that is involved in many biological processes, such as plant growth, development, and environmental stress (Chuck et al., 2002; Aharoni et al., 2004; Broun et al., 2004; Mizoi, Shinozaki & Yamaguchi-Shinozaki, 2012). Each AP2/ERF contains the AP2/ERF conserved domain that consists of 60–70 amino acid residues, which results in the name for the AP2/ERF family. The AP2 domain regulates the expression of target genes by binding to the GCC-box (Ohme-Takagi & Shinshi, 1995), the dehydration responsive element (DRE) (Sun et al., 2008; Guttikonda et al., 2014), and/or the TTG element (Wang et al., 2015). The AP2/ERF family is divided into three subfamilies (AP2, ER, and RAV) based on the similarity of amino acid sequences and number of conserved domains (Nakano et al., 2006). There are two AP2/ERF domains in the AP2 subfamily, one AP2/ERF and one B3 domain in the RAV subfamily, and one AP2/ER domain in the ERF subfamily. In addition, the ERF subfamily is divided into ER and CBF/DREB subgroups, with differences at the 14th and 19th amino acid (Sakuma et al., 2006).

AP2/ERF family members have been isolated, and their functions have been identified in many species (Xu et al., 2011; Mizoi, Shinozaki & Yamaguchi-Shinozaki, 2012; Licausi, Ohme-Takagi & Perata, 2013). Overexpression of members of the subfamily DREB in transgenic plants increased resistance to abiotic stress, such as drought (Hong & Kim, 2005; Oh et al., 2009; Fang et al., 2015), salt (Hong & Kim, 2005; Bouaziz et al., 2013), cold (Fang et al., 2015), and high temperatures (Qin et al., 2007). Also, the overexpression of ERF members not only improved the resistance to multiple biological stresses by regulating the expression of defense genes (Berrocal-Lobo, Molina & Solano, 2002; Guo et al., 2004; Dong et al., 2010; Moffat et al., 2012), but also increased resistance to abiotic stress, such as drought (Zhang et al., 2010a; Zhang et al., 2010b; Yang et al., 2016), high salt concentrations (Guo et al., 2004), freezing (Zhang & Huang, 2010), and osmotic stress (Zhang et al., 2010a). Members of the AP2 subfamily played important roles in the development of flowers, fruits, and seeds (Maes et al., 2001; Jofuku et al., 2005; Chung et al., 2010; Horstman et al., 2014). RAV members were responded to ethylene, brassinolide (BR), and biotic and abiotic stress (Mittal et al., 2014).

Apple (*Malus* × *domestica* Borkh.) is one of the most important tree fruits in the world. However, progress on the ERF transcription factors in apple is more limited than that in model plants like *Arabidopsis thaliana*, and most researches about apple are focused on fruit ripening and softening (Wang et al., 2007; Tacken et al., 2010; Li et al., 2016; An et al., 2017; Li et al., 2017; Han et al., 2018a; Han et al., 2018b). In this study, we obtained the AP2/ERF transcription factor in apple based on previous results (Girardi et al., 2013) and from the Plant Transcription Factor Database (http://planttfdb.cbi.pku.edu.cn/). When the 60 known transcription factors were excluded by sequence alignment in the GenBank database (Tacken et al., 2010), the other genes were cloned and analyzed. In total, 30 genes in the AP2/ERF family were obtained. Furthermore, we analyzed the phylogenetic relationships, subcellular locations, and expression levels in different tissues under different biotic and abiotic stresses for the 30 AP2/ERF genes. The results are helpful for further studying roles of AP2/ERF transcription factors played in growth, development, and biotic and abiotic stress in apple.

## Materials & Methods

### Plant materials

The apple cultivar ‘Gala’ (*Malus* × *domestica* cv. Gala) was used as material under stress conditions. *In vitro* seedlings of ‘Gala’ were cultivated on basic subculture medium (MS medium + 0.2 mg L^−1^ indole-3-acetic acid (IAA) + 0.8 mg L^−1^ 6-benzylaminopurine (6-BA) + 30 g L^−1^ sucrose + 7 g L^−1^ agrose) that was changed every 30 d. The cultivation conditions were under 14-h light/10-h dark and a temperature of 24  ± 2 °C. On the 20th day on the basic subculture medium, some relatively uniform seedlings were selected and transplanted to different media. The basic subculture medium was used as the control. We added 150 mmol L^−1^ NaCl or 300 mmol L^−1^ mannitol to the basic subculture medium to create different treatments (Li et al., 2019).

### Gene cloning and sequence analysis

RNA was extracted in the fully expanded leaves of ‘Zihong Fuji’ apple (*Malus* × *domestica* cv. Zihong Fuji) by the CTAB method, then cDNA was synthesized using a PrimeScript™ II 1st Strand cDNA Synthesis Kit (Takara, Dalian, China). Based on the nucleotide sequence of 259 identified members in the apple AP2/ERF gene family and the 60 known transcription factors in the GenBank database (Tacken et al., 2010; Velasco et al., 2010; Girardi et al., 2013), we designed primers for PCR amplification and 30 apple *AP2/ERF* genes were finally cloned (Table S1). The PCR reaction conditions were 94 °C for 5 min, then 35 cycles for 94 °C for 1 min 20 s, 56–60 °C for 1 min, 72 °C for 2 min, and a final extension at 72 °C for 10 min. PCR products were purified and cloned into pMD19-T vector to construct recombinant plasmids. The recombinant plasmids were transformed into the competent cells of *Escherichia coli* DH5α, and then the positive clones were selected.

The cDNA sequences that we obtained were used as queries in BLASTN searches against NCBI (https://www.ncbi.nlm.nih.gov/). The open reading frame (ORF) and amino acid sequences were analyzed by DNAMAN 6.0 software. The phylogenetic tree was constructed by MEGA 6 software according to the unrooted Neighbour Joining (NJ) method with execution parameters: the Poisson correction, pairwise deletion, and bootstrap (1,000 replicates), using full-length amino acid sequences from AP2/ERF proteins of apple and *Arabidopsis*. The conserved domains were predicted by Pfam 26.0 (http://pfam.xfam.org/) and the Conserved Domains program in NCBI (https://www.ncbi.nlm.nih.gov/Structure/cdd/wrpsb.cgi). CELLO v.2.5 (http://cello.life.nctu.edu.tw/), PSORT (https://psort.hgc.jp/form.html), and SoftBerry ProtComp 9.0 (http://linux1.softberry.com/) were used to predict subcellular locations (Dong et al., 2018a; Dong et al., 2018b; Dong et al., 2018c; Hao & Qiao, 2018).

### Subcellular localization analysis

The full-length cDNA without the stop codon of *MdERF28* was introduced into the pCAMBIA2300-GFP vector. The fusion vectors were then introduced into *Agrobacterium tumefaciens* strain EHA105 and then infiltrated into tobacco leaves. Those infected tissues were analyzed 72 h after infiltration, under a fluorescence microscope (BX63; Olmypus, Tokyo, Japan).

### Gene expression analysis

The expression data for the AP2/ERF gene family in different tissues were obtained at Gene Expression Omnibus (GEO, https://www.ncbi.nlm.nih.gov/geo/) with GEO accession number GSE42873 (Celton et al., 2014). These existing data included a set of expression arrays from 16 different apple tissues (from 10 different genotypes of apple: leaf_M14 (fully developed), fruit_M20_100 DAM (100 days after anthesis)/_harvest (harvested at maturity), leaf_M49 (fully developed), flower_M67, flower / fruit _M74_100 DAM/_Harvest, root (growing root tip)/ stem (fully developed)/ seedling (10 days old)_GD, seedling (10 days old)_X4102, root (growing root tip)/ stem (fully developed)_X8877, seed (dormant seed)_X4442 × X2596 and seed (dormant seed)_X3069 × X922), with two biological replicates for each tissue, and a known array probe was used as the MDP identification number in apple genome database V1.0. The RNA-seq data for *AP2/ERF* response to AAAP was from Zhu et al. (2017).

The RNA was extracted from the treated tissues of ‘Gala’ using a RNeasy Plant Mini Kit (QIAGEN, China, Item No. 74903), and the cDNA was synthesized using the PrimeScript™ II 1st Strand cDNA Synthesis Kit (Takara, Dalian, China). The qRT-PCR primers (Table S1) were designed based on the 3′- or 5′-UTR of *AP2/ERF* genes, and then qRT-PCR was conducted using a 3-step method by BIO-RAD IQ5 (USA) with *MdMDH RNA* as the internal reference gene (Perini et al., 2014). Three independent biological replicates were used for calculations. Each 20 µL qRT-PCR reaction mixture consisted of SYBR Green Master I 10 µL, 5 µmol L^−1^ forward prime 1 µL, 5 µmol L^−1^ reverse prime 1 µL, template 1 µL, and ddH_2_O 7 µL. qRT-PCR conditions were 95 °C for 3 min, then 40 cycles for 95 °C for 10 s, 58.5 °C for 30 s, 72 °C for 15 sand, after annealing to 55 °C, the temperature was increased 0.5 °C every 7 s till 95 °C, with 81 cycles in total. The 2^−ΔΔ*CT*^ method was used to analyze the data (Livak & Schmittgen, 2001).

## Results

### Cloned genes in the AP2/ERF family in apple

**Figure 1:**
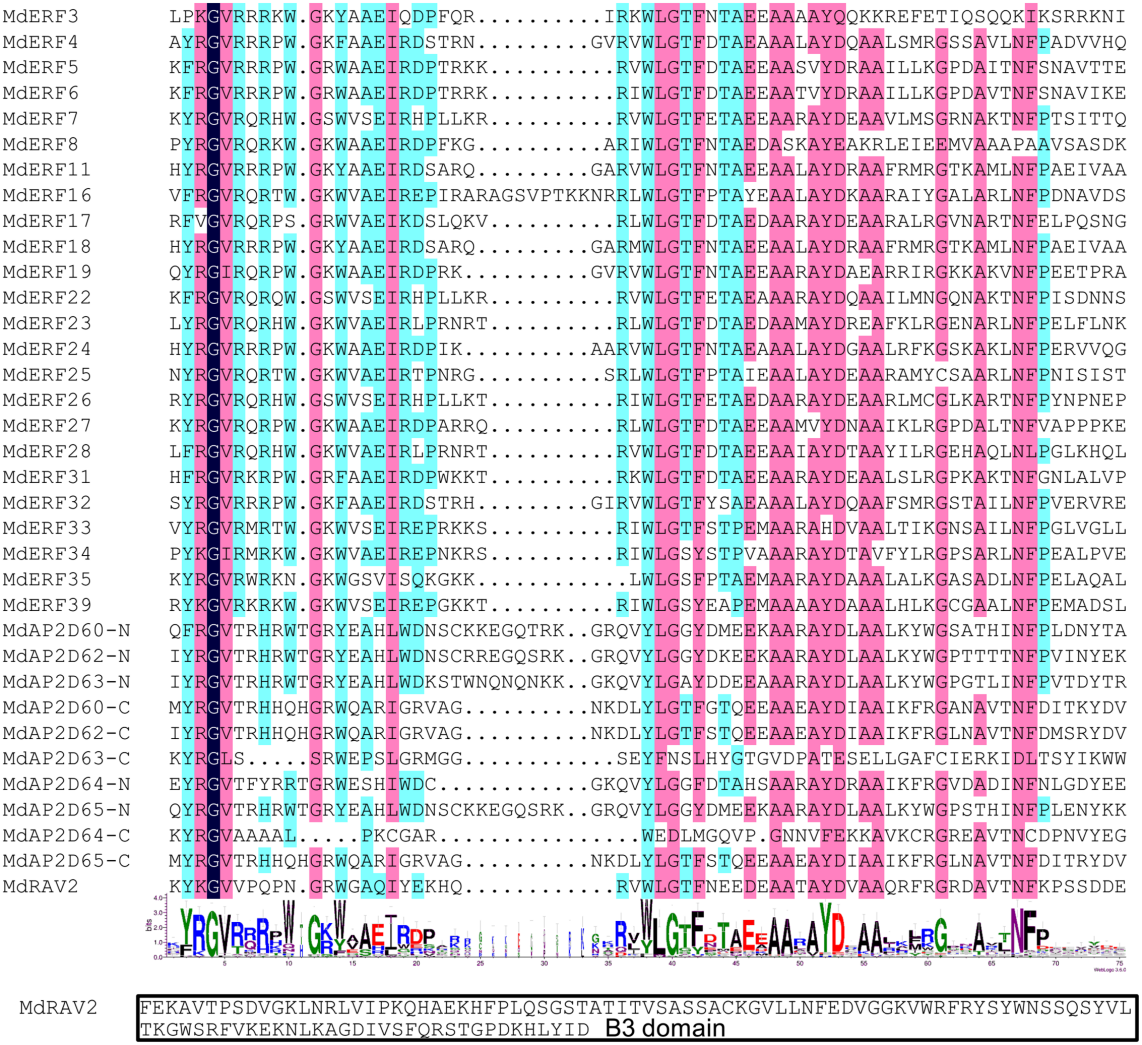
Sequence analysis of the AP2 and B3 domain in apple AP2/ERF proteins. The AP2 and B3 domains were reconstructed based on the alignment of the apple conserved AP2 and B3 regions. Sequence alignment was generated by DNAMAN 6.0 software. Sequence logo was built by online software WebLogo 3.0. The heights of symbols within each stack indicate the relative frequency of each amino acid at that position.

Based on the nucleotide sequence of 259 identified members in the apple AP2/ERF gene family and the 60 known transcription factors in the GenBank database (Tacken et al., 2010; Velasco et al., 2010; Girardi et al., 2013), the other primers for PCR amplification were designed, and a total of 30 genes in the apple AP2/ERF family were cloned (Table 1). Homology alignment for the amino acid information showed that all the MdAP2/ERF proteins contained an AP2 conserved domain (Fig. 1). Both MdAP2D60 and MdAP2D62-65 had two AP2 conserved domains, and MdRAV2 had one B3 conserved domain (Fig. 1).

**Table 1:** The AP2/ERF genes in apple.

Gene name	V1.0 gene ID^a^	GDDH13 gene ID^b^	GeneBank accession	GDDH13 Chromosome location	ORF	Amino acid	MW	PI	Group
*MdERF3*	MDP0000119204	MD14G1226300	MG099812	Chr14:30769541-30771506	1029	342	38.731	4.763	B6
*MdERF4*	MDP0000322279	MD04G1228800	MG099813	Chr04:30908814-30909560	747	248	27.933	4.795	B3
*MdERF5*	MDP0000464704	MD11G1052100	MG099814	Chr11:4448529-4449446	918	305	33.478	6.05	B5
*MdERF6*	MDP0000190504	MD03G1049900	MG099815	Chr03:3981184-3982008	825	274	30.132	7.242	B5
*MdERF7*	MDP0000290880	MD15G1172600	MG099816	Chr15:13418043-13419309	708	235	26.092	9.006	B6
*MdERF8*	MDP0000759299	MD16G1043500	MG099817	Chr16:3058483-3060008	1032	343	38.168	4.514	B6
*MdERF11*	MDP0000290585	MD17G1089700	MG099820	Chr17:7361577-7363808	594	197	21.478	5.857	A2
*MdERF16*	MDP0000153866	MD04G1165400	MG099825	Chr04:25593675-25596164	1476	491	55.192	4.949	A2
*MdERF17*	MDP0000127123	MD06G1125700	MG099826	Chr06:26773748-26774617	870	289	31.312	5.64	B6
*MdERF18*	MDP0000246184	MD04G1009000	MG099827	Chr04:1044443-1045735	1293	430	47.099	9.09	A6
*MdERF19*	MDP0000308922	MD17G1152400	MG099828	Chr17:14092036-14095154	1164	387	42.728	4.698	B2
*MdERF22*	MDP0000287350	MD15G1124900	MG099831	Chr15:9070265-9071680	642	213	23.903	6.365	B6
*MdERF23*	MDP0000764803	MD17G1244300	MG099832	Chr17:29279809-29281131	1338	445	49.63	7.101	A6
*MdERF24*	MDP0000190237	MD14G1147100	MG099833	Chr14:23978106-23980121	630	209	23.033	9.918	B4
*MdERF25*	MDP0000689946	MD10G1286300	MG099834	Chr10:37571420-37572034	615	204	22.67	9.731	B3
*MdERF26*	MDP0000279733	MD17G1220600	MG099835	Chr17:26952408-26953440	429	142	15.885	5.821	B6
*MdERF27*	MDP0000854039	MD01G1214500	MG099836	Chr12:30790168-30791244	750	249	27.666	3.996	B5
*MdERF28*	MDP0000805422	MD05G1306900	MG099837	Chr05:43876234-43876827	573	190	20.896	9.165	B3
*MdERF31*	MDP0000457509	MD10G1191300	MG099840	Chr10:28815601-28816149	549	182	20.062	10.012	B1
*MdERF32*	MDP0000235313	MD16G1216900	MG099841	Chr16:21318271-21318960	555	184	20.958	6.433	B3
*MdERF33*	MDP0000652413	MD02G1060200	MG099842	Chr02:4815948-4816775	828	275	29.869	4.844	A4
*MdERF34*	MDP0000125673		MG099843		477	158	17.134	8.467	A5
*MdERF35*	MDP0000228713	MD07G1099500	MG099844	Chr07:10964414-10968649	1203	400	44.015	7.624	A4
*MdERF39*	MDP0000122739	MD15G1396500	MG099848	Chr15:49614193-49614798	606	201	22.232	5.245	A5
*MdAP2D60*	MDP0000187703	MD15G1064600	MG099849	Chr15:4496397-4499611	1956	651	71.47	7.209	AP2
*MdAP2D62*	MDP0000121984	MD13G1252700	MG099851	Chr13:27049619-27053293	1668	555	60.599	8.049	AP2
*MdAP2D63*	MDP0000314518	MD12G1075200	MG099852	Chr12:9108198-9111657	1407	468	50.657	8.133	AP2
*MdAP2D64*	MDP0000281079	MD01G1113400	MG099853	Chr01:22726643-22730520	1275	424	46.804	8.297	AP2
*MdAP2D65*	MDP0000801540	MD02G1190000	MG099854	Chr02:17483494-17487257	1962	653	72.224	7.145	AP2
*MdRAV2*	MDP0000939633	MD16G1047700	MG099860	Chr16:3329564-3330772	1206	401	43.821	9.241	RAV

**Notes.**

a V1.0 gene ID represents gene ID from apple V1.0 database (Velasco et al., 2010).

b GDDH13 gene ID represents gene ID from apple GDDH13 v1.1 database.

### Phylogenetic analysis of AP2/ERF proteins in apple

**Figure 2:**
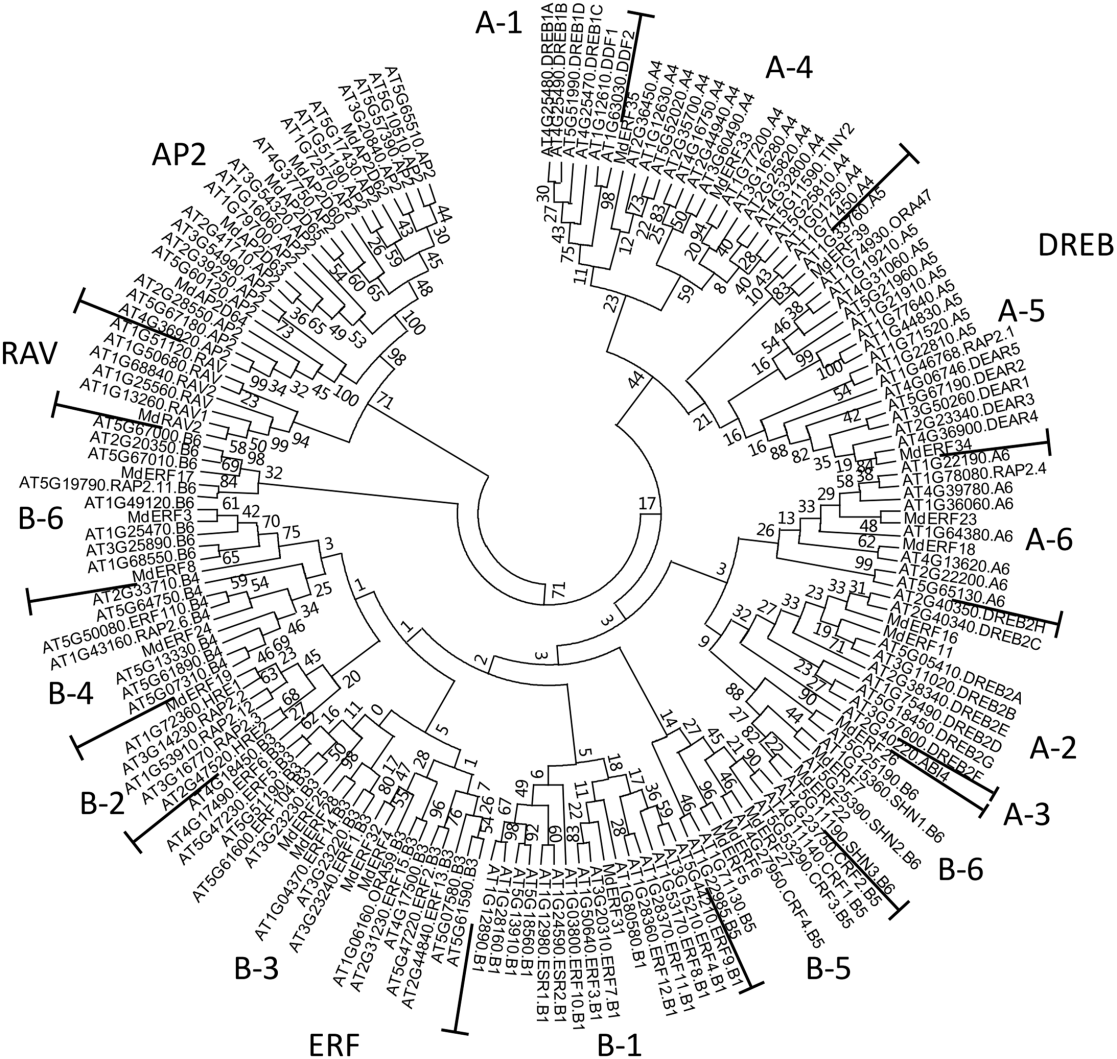
Phylogenetic relationships and subfamily classification of AP2/ERF proteins from apple and *Arabidopsis*. Unrooted Neighbour Joining (NJ) phylogenetic tree was constructed with MEGA 6 software using full-length amino acid sequences from AP2/ERF proteins of apple and *Arabidopsis*. The tree was classified into four subfamilys (DREB: A1–A6, ERF: B1–B6, AP2 and RAV).

The MdAP2/ERF proteins were clustered and analyzed using MEGA6 software, and the known MdAP2/ERF protein types in *Arabidopsis thaliana* were used to identify the type of apple AP2/ERF protein. There were four subfamilies, DREB, ERF, RAV, and AP2 in the apple AP2/ERF protein family; DREB included groups A-1, A-2, A-3, A-4, A-5, and A-6, and ERF contained groups B-1, B-2, B-3, B-4, B-5 and B-6. Further, proteins MdERF11/16, MdERF33/35, MdERF34/3, and MdERF18/23 were clustered into groups A-2, A-4, A-5, and A-6 in the DREB subfamily, respectively. MdERF31, MdERF19, MdERF4/25/28/32, MdERF24, MdERF5/6/27, and MdERF3/7/8/17/22/26 were clustered into groups B1, B-2, B-3, B-4, B-5, and B-6 in the ERF subfamily, respectively. Proteins MdAP2D60 and MdAP2D62-MdAP2D65 were clustered into the AP2 subfamily; MdRAV2 was clustered into the RAV subfamily (Fig. 2, Table 1).

### Subcellular locations of AP2/ERF proteins in apple

**Figure 3:**
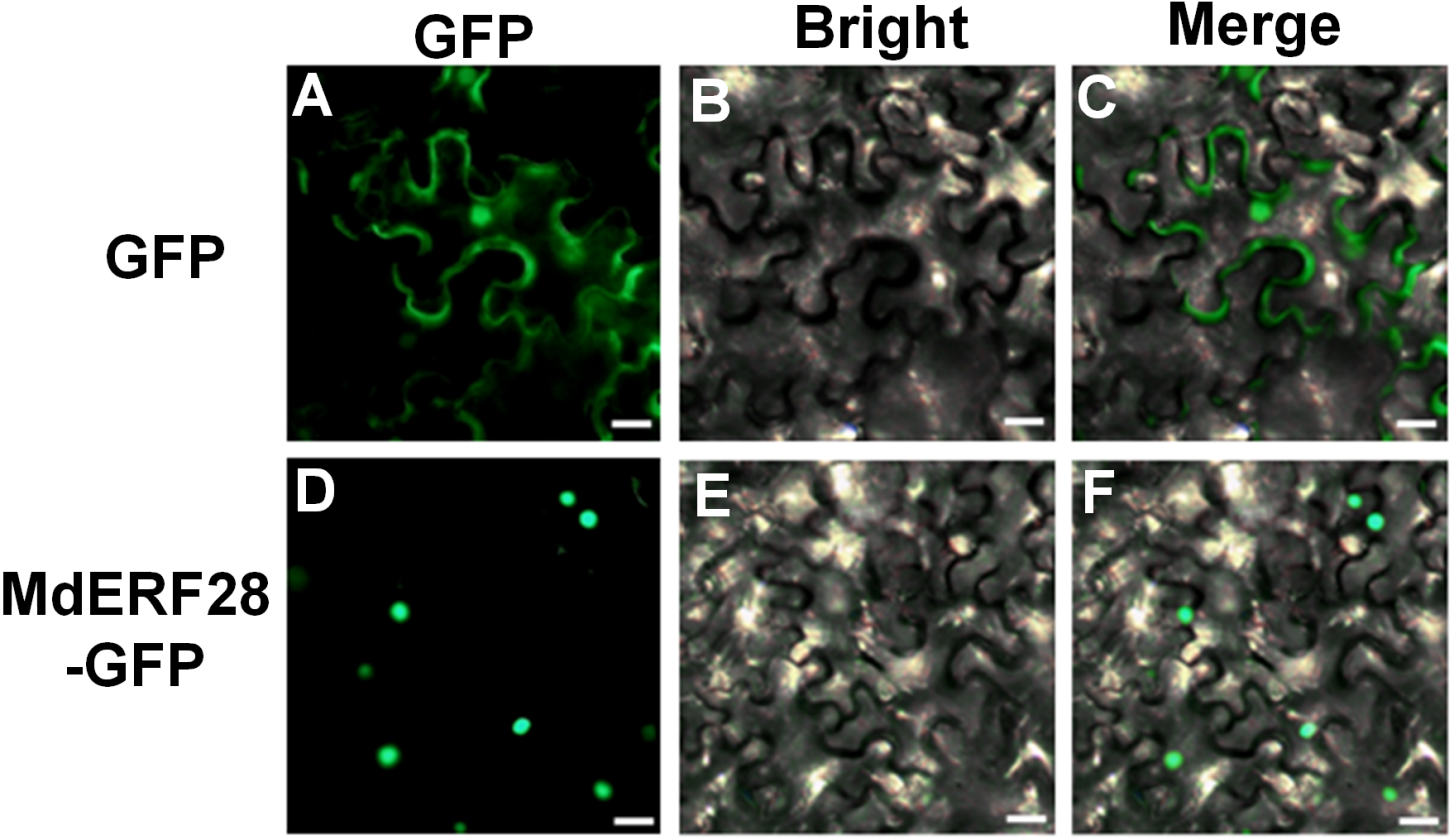
Subcellular localization assay of the MdERF28 protein. (A) Fluorescence microscopy image of GFP; (B) bright-field image of GFP; (C) GFP merged image; (D) fluorescence microscopy image of MdERF28-GFP; (E) Bright-field image of MdERF28-GFP; (F) MdERF28-GFP merged image. Scale bar = 50 mm.

Subcellular localization of AP2/ERF proteins was performed by SoftBerry ProtComp 9.0, CELLO, and PORST using their protein sequences. All prediction results indicated that MdERF3-8, MdERF11, MdERF16-19, MdERF22-28, MdERF33-35, MdERF39, MdAP2D60, MdAP2D62-65, and MdRAV2 were target to nuclear (Table 2). To further verification of these subcellular locations revealed by the online software, the MdERF28-GFP fusion protein was performed to detect the subcellular location of MdERF28 protein and a transient transfection assay into tobacco leaves. The GFP control was ubiquitously distributed throughout the cell, whereas MdERF28-GFP fusion protein was predominantly detected in the nucleus (Fig. 3), indicating that MdERF28 was localized in the nucleus.

### Expression analysis of 30 AP2/ERF gene family in apple

**Figure 4:**
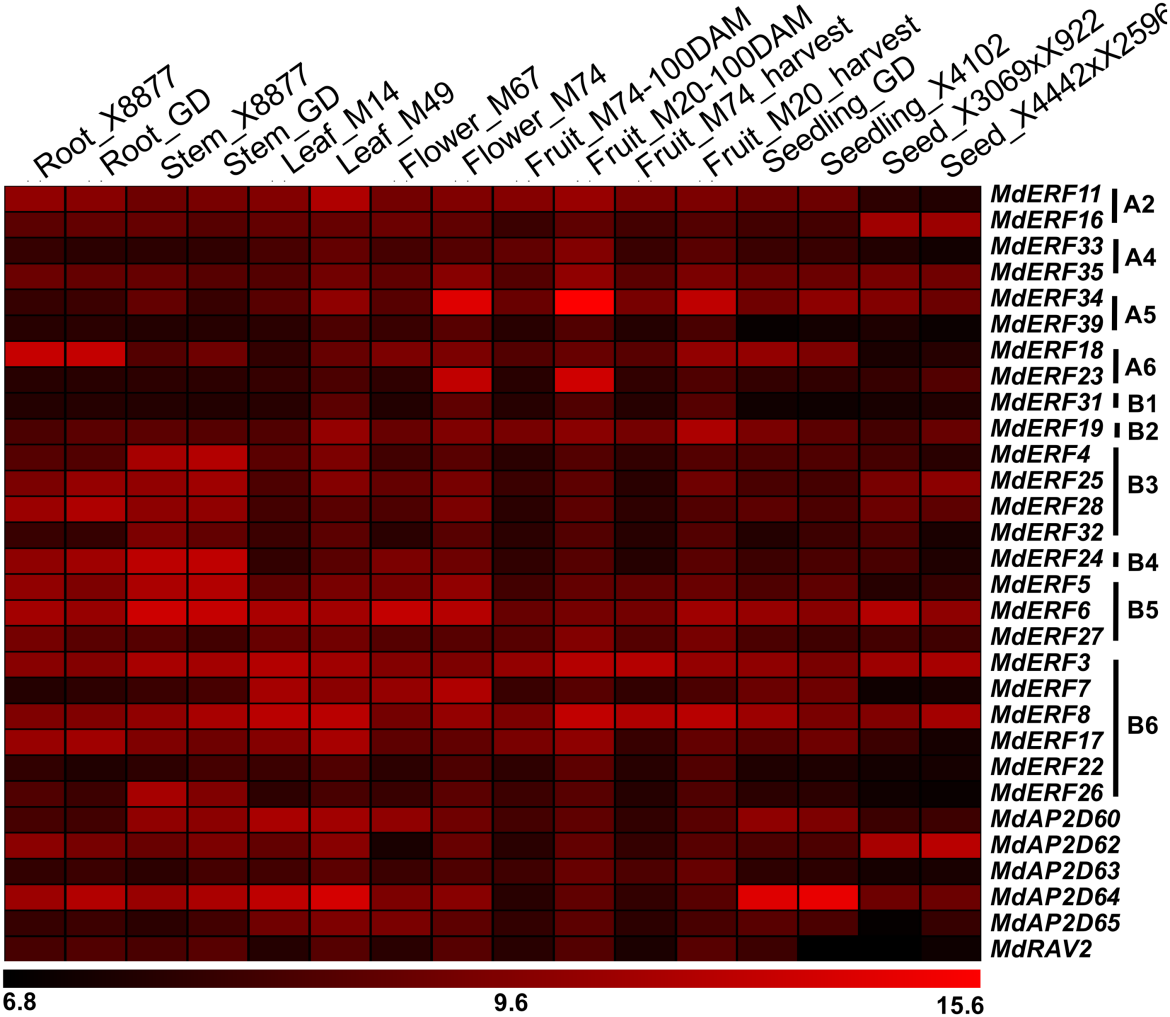
Expression profiles of apple *AP2/ERF* genes in various tissues. The data of apple *AP2/ERF* expression (GSE42873) in 16 different were searched at GEO database in NCBI. The heat map of apple *AP2/ERF* genes was generated by TIGR MeV v4.8.1 software.

The array (GSE42873) in 16 different apple tissues in GEO (https://www.ncbi.nlm.nih.gov/geo/) was used to evaluate the expression level of the AP2/ERF gene family in different tissues (Fig. 4). The 30 *AP2/ERF* genes exhibited diverse expression patterns among the various tissues (Fig. 4).

**Figure 5:**
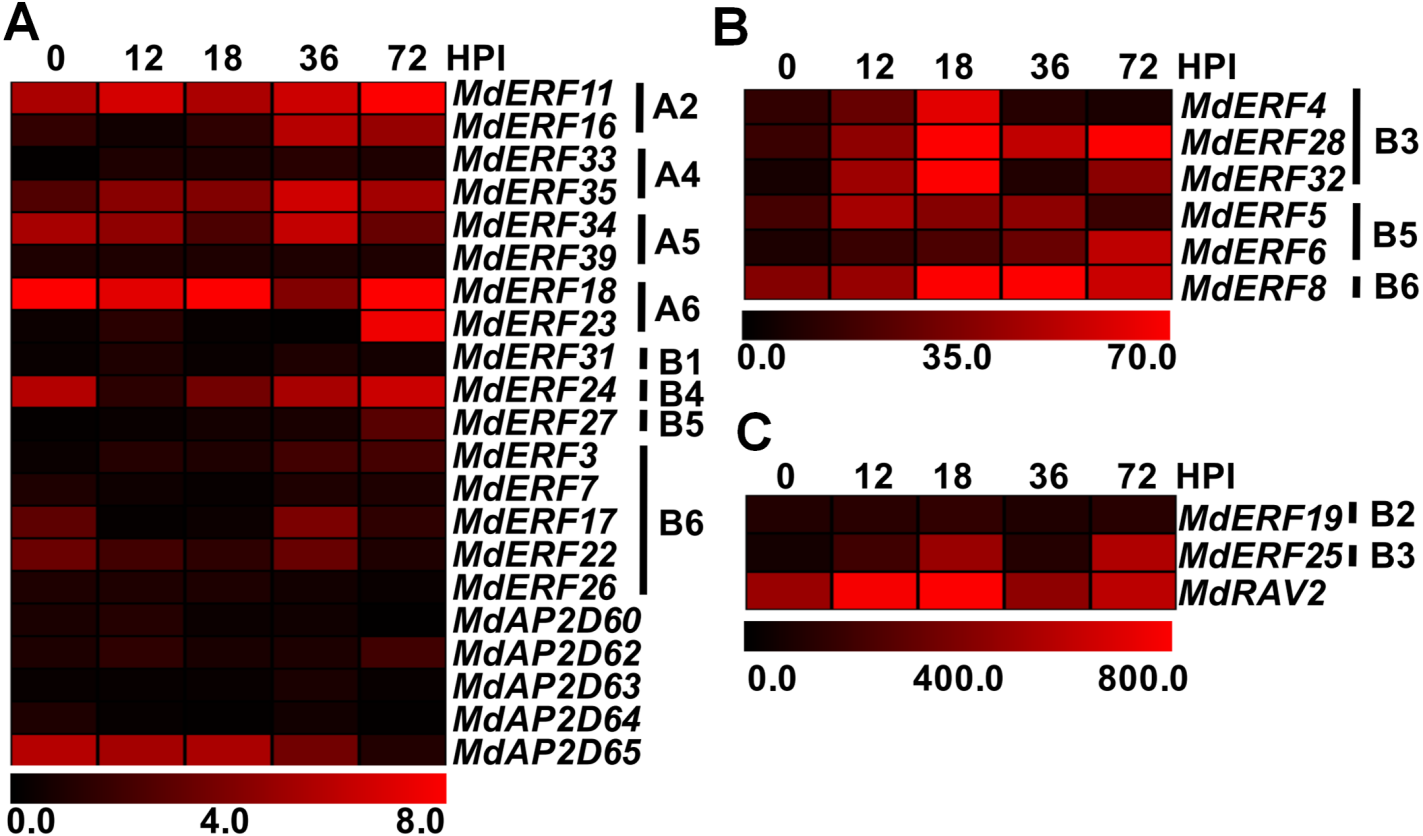
Expression profiles of apple *AP2/ERF* genes in response to *Alternaria alternata* apple pathotype infection. (A) Expression profiles of 21 apple *AP2/ERF* genes in response to AAAP infection; (B) expression profiles of five apple *AP2/ERF* genes in response to AAAP infection; (C) expression profiles of three apple *AP2/ERF* genes in response to AAAP infection. The expression data of apple *AP2/ERF* genes in response to AAAP infection were obtained from supplementary data previously published study (Zhu et al., 2017). The heat map of apple *AP2/ERF* genes was generated by TIGR MeV v4.8.1 software.

Further, we detected the expression level of the response of the AP2/ERF gene family to AAAP infection using RNA-seq with existing data (>two-fold and FDR<0.001) (Zhu et al., 2017). *MdERF16* in A2, *MdERF35* in A4, *MdERF23* in A6, *MdERF25/28/32* in B3, *MdERF6/27* in B5, and *MdERF8* in B6 were all up-regulated in the response of apples’ AAAP infection (Fig. 5 and File S3). Particularly, the expression level of B3 in *MdERF32* was increased significantly, which was 12.6-folds by 18 h post inoculation (HPI). Expression levels of *MdERF23* in A6, *MdERF25* in B3, *MdERF28* in B3, and *MdERF27* in B5 were all increased, which were 18.2, 8.4, 16.2, and 8.7-fold by 72 HPI, respectively. During the early (12 HPI) and intermediate (18 and 36 HPI) phase of infection, expression levels of *MdERF4* in B3 and *MdERF5* in B5 were increased at the beginning and then decreased later, and expression of *MdERF4* was 4.6-fold by 18 HPI. Expression levels of *MdERF22* in B6 and *MdAP2D65* was down-regulated on 72 HPI (Fig. 5 and File S3). The relative expression level of other genes did not change significantly (Fig. 5 and File S3).

**Table 2:** The information in predicting apple AP2/ERF subcellular localization.

Location	Nuclear	Plasma membrane	Extracellular	Cytoplasmic	Mitochondrial	Endoplasm. retic	Peroxisomal	Golgi	Chloroplast	Vacuolar
MdERF3	4.41	1.16	0	2.19	1.25	0.25	0.53	0	0	0.2
MdERF4	9.99	0	0	0	0	0	0	0	0.01	0
MdERF5	9.99	0.01	0	0	0	0	0	0	0	0
MdERF6	9.99	0	0	0	0	0	0	0	0.01	0
MdERF7	9.99	0	0	0	0	0	0	0	0.01	0
MdERF8	6.04	0.34	0.27	0.35	2.43	0.16	0	0	0.41	0
MdERF11	10	0	0	0	0	0	0	0	0	0
MdERF16	4.9	0.53	0.18	2.43	1.34	0.28	0.26	0	0	0.08
MdERF17	5.61	0.36	1.04	1.08	1.44	0	0.33	0.04	0.1	0
MdERF18	9.97	0	0	0	0	0	0	0	0.03	0
MdERF19	9.98	0	0	0	0	0	0	0	0.02	0
MdERF22	9.96	0.04	0	0	0	0	0	0	0	0
MdERF23	9.46	0	0.03	0.3	0.2	0	0	0	0	0.01
MdERF24	9.98	0.02	0	0	0	0	0	0	0	0
MdERF25	9.99	0	0	0	0	0	0	0	0	0
MdERF26	9.99	0.01	0	0	0	0	0	0	0	0
MdERF27	4.35	0.98	0.64	0.16	3.3	0.1	0.22	0	0.23	0.02
MdERF28	10	0	0	0	0	0	0	0	0	0
MdERF31	9.9	0	0	0	0	0	0	0	0.09	0.01
MdERF32	9.97	0	0	0	0	0	0	0	0.03	0
MdERF33	9.99	0	0	0	0	0	0	0	0.01	0
MdERF34	9.99	0	0	0	0	0	0	0	0	0
MdERF35	5.69	1.01	0.43	1.1	1.09	0	0	0	0.69	0
MdERF39	9.99	0	0	0	0	0	0	0	0	0
MdAP2D60	10	0	0	0	0	0	0	0	0	0
MdAP2D62	10	0	0	0	0	0	0	0	0	0
MdAP2D63	9.99	0	0.01	0	0	0	0	0	0	0
MdAP2D64	9.98	0	0	0	0	0	0	0	0.02	0
MdAP2D65	10	0	0	0	0	0	0	0	0	0
MdRAV2	9.99	0	0.01	0	0	0	0	0	0	0

**Figure 6:**
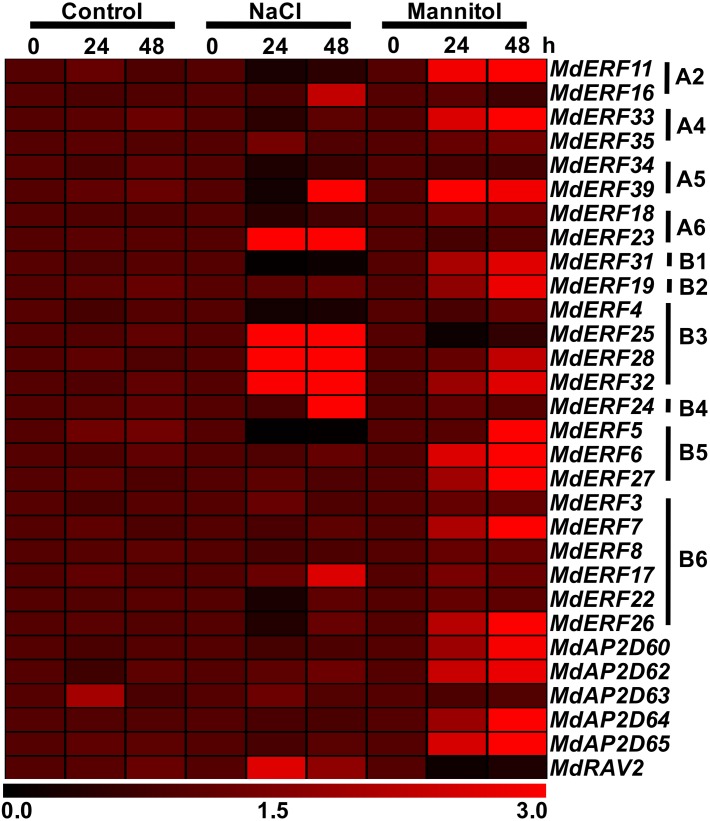
Expression heatmap of apple *AP2/ERF* genes under normal growth, mannitol and salt treatments. The expression data of apple *AP2/ERF* genes under normal growth, mannitol and salt treatments were obtained from qRT-PCR. The heat map of apple *AP2/ERF* genes was generated by TIGR MeV v4.8.1 software.

The AP2/ERF gene family expression in ‘Gala’ seedlings under mannitol and NaCl stress was analyzed by qRT-PCR. Under NaCl stress, eight members in the AP2/ERF family were up-regulated, which included *MdERF16* in A2, *MdERF23* in A6, *MdERF25/28/32* in B3, *MdERF24* in B4, *MdERF17* in B6, and *MdRAV2* (Fig. 6). Among them, the expression level of *MdERF23*, *MdERF25*, and *MdERF28* were increased more than 10 times when treated for 48 h compared with that of the control. *MdERF11* in A2, *MdERF33* in A4, *MdERF34* in A5, *MdERF18* in A6, *MdERF31* in B1, *MdERF4* in B3, *MdERF5* in B5, and *MdERF22/26* in B6 were down-regulated. Expression levels of *MdERF5* and *MdERF39* were only 0.04 and 0.23 times that of the control, respectively, when treated with NaCl for 24 h, but expression level of *MdERF39* was increased to 3.11 times that of the control when treated for 48 h. The other AP2/ERF genes under NaCl stress had almost the same expression level compared with that of the control (Fig. 6).

Under mannitol treatment condition, the relative expression levels of *MdERF11* (A2), *MdERF33* (A4), *MdERF39* (A5), *MdERF31* (B1), *MdERF19* (B2), *MdERF28/32* (B3), *MdERF5/6/27* (B5), *MdERF7* (B6), and *MdAP2D60/62/64/65* were increased compared with the control, *MdERF39* (A5) reached 5.96 times that of the control at 24 h, and *MdERF11* (A2) and *MdAP2D65* reached 7.99 and 10.7 times that of the control, respectively, when treated for 48 h (Fig. 6).The relative expression level of *MdERF25* (B3) and *MdRAV2* were inhibited compared with that of the control, but the relative expression level of other AP2/ERF genes did not change significantly under mannitol treatment (Fig. 6).

## Discussion

Based on the draft genome sequence of the domesticated apple (*Malus* × *domestica*) and the highly conserved domain in the AP2/ERF transcription factors of plants, 259 genes in the apple AP2/ERF family were selected for analyzing ERF transcription factors in apple genome database ver 1.0 (Velasco et al., 2010; Girardi et al., 2013). In this study, we cloned 30 apple *AP2/ERF* genes, which belonged to the AP2, ERF, DREB, and RAV subfamilies of AP2/ERF, and their changes in expression level in different tissues were analyzed under AAAP infection, and NaCl and mannitol stresses.

ERF is one the largest transcription factor families in plants. The *A. thaliana* genome contained 147 AP2/ERF proteins, which were divided into the AP2, ERF (ERF and DREB), and RAV sub-families based on their similarity in amino acid sequences and domain number (Nakano et al., 2006; Mizoi, Shinozaki & Yamaguchi-Shinozaki, 2012). The 30 genes cloned in this study were divided into four subfamilies; 8, 16, 5, and 1 gene belonged to the subfamilies DREB, ERF, AP2, and RAV, respectively (Fig. 2). AAAP infection, NaCl stress, and mannitol stress all affected the expression of *MdERF4/25/28/32* in the B3 group at transcriptional level, except for *MdERF4* under mannitol stress. In *A. thaliana*, *AtERF1*, *AtERF2*, *AtERF5*, and *AtERF6* in the B3 group, which could be induced by osmotic stress (Moffat et al., 2012), were responded to *Saprophytic bacteria* by up-regulating the downstream resistance genes *PDF1.2* and *b-CHI*, resulting in enhanced resistance to *S. bacteria* infection (Fujimoto et al., 2000; Berrocal-Lobo, Molina & Solano, 2002; Lorenzo et al., 2003; Moffat et al., 2012). Alfafa exhibited increased resistance from *MtERF1-1* in the B3 group that up-regulated the resistance downstream gene *PDF1.2* (Anderson et al., 2010). In wheat, *TaPIEP1* in the B3 group was up-regulated by *Bipolaris sorokiniana*, which boosting disease resistance (Dong et al., 2010). Transgenic tobacco plants had enhanced resistance to Tobacco Mosaic Virus and brown spot through overexpression of *NtERF5* and *GbERF2* (Fischer & Droge-Laser, 2004; Zuo et al., 2007). The transgenic *A. thaliana* with *SpERF1*, which was the ERF member of the B3 group in *Stipa purpurea*, had increased drought tolerance when *SpERF1* was up-regulated (Yang et al., 2016). In this study, MdERF4/25/28/32 was clustered into the B3 group of ERF and was up-regulated significantly under AAAP infection and NaCl stress; also, mannitol stress had some effects on *MdERF4/25/28/32* expression (Figs. 5 and 6). These results indicated that MdERF4/25/28/32 may play important roles in response to various biotic and abiotic stress.

Several studies have proved that the DREB transcription factor subfamily was important for abiotic stress (Nakano et al., 2006). For example, *A. thaliana* showed increased tolerance to high-salt and drought by overexpression of certain DREB transcription factors that included *DREB2A* and *DREB2B* in the A2 group, *HARDY* in the A4 group, and *RAP2.4* in the A6 group. *DREB2C*, *DREB2D*, and *DREB2F* in *A. thaliana* played an important role in high-salt stress (Nakano et al., 2006; Sakuma et al., 2006; Karaba et al., 2007; Qin et al., 2007; Lin, Park & Wang, 2008). Drought tolerance in maize was enhanced by *DREB2A* overexpression in the A2 group (Qin et al., 2007). Overexpression of the *PsAP2* gene in the A6 group of *Papaver somniferum* enhanced the resistance of transgenic tobacco to pathogenic bacteria, salt, and mannitol stresses (Mishra et al., 2015). In this study, under mannitol stress, *MdERF11* in the A2 group, *MdERF33* in the A4 group, and *MdERF39* in the A5 group were up-regulated at transcriptional level. Seven genes were induced by NaCl at transcriptional level. Three of them, *MdERF16* in A4, *MdERF39* in A5, and *MdERF23* in A6, were up-regulated at transcriptional level under NaCl stress, and four genes, which included *MdERF11* in A2, *MdERF33* in A4, *MdERF34* in A5, and *MdERF18* in A6, were down-regulated. In addition, there were four genes, which included *MdERF16* in A2, *MdERF35* in A4, and *MdERF23* in A6, were up-regulated at transcriptional level by AAAP infection (Figs. 5 and 6). These results showed that the DREB transcription factors cloned in this study might be important for responding to abiotic stress, and some members might play a role in response to biotic stress.

The AP2 subfamily may be important for plant growth and development (Maes et al., 2001; Jofuku et al., 2005; Chung et al., 2010; Horstman et al., 2014), but also be critical for defending against biotic and abiotic stress (Park et al., 2001; Yi et al., 2004). For example, the overexpression of the *Tsi1* gene improved tobacco’s tolerance to pathogenic bacteria and osmotic stress (Park et al., 2001), and the *CaPF1* gene in *Capsicum annuum* cv. Bukang responded to ethylene (ET), jasmonic acid (JA), and cold stress, and its overexpression improved *A. thaliana* resistance to low temperature and to infection by *Pseudomonas syringae* pv. tomato DC3000 (Yi et al., 2004). In this study, *MdAP2D65* in AP2 responded to AAAP infection only at transcriptional level, but it did not respond to NaCl stress, and *MdAP2D60/62/64/65* were up-regulated by mannitol stress (Figs. 5 and 6). These results indicated that *MdAP2D60/62/64/65* had some effect on osmotic stress, and *MdAP2D65* might be involved in responding to biotic stress.

## Conclusions

Thirty novel *AP2/ERF* genes have been successfully isolated from *Malus domestica*, which belong to DREB, ERF, AP2, and RAV subfamily. Results of a known array and RNA-seq analysis using existing data as well as qRT-PCR-based transcription profiling indicated that 30 apple *AP2/ERF* genes were expressed in all examined tissues at different expression levels, and responded differentially to various stresses, suggesting that these genes may be involved in the regulation of growth, development, and stress responses in apple. These results serve as the theoretical basis for understanding the biological function and regulation of AP2/ERF transcription factors in apple.

##  Supplemental Information

10.7717/peerj.8391/supp-1 File S1 cDNA sequences of 30 apple AP2/ERF TFs

10.7717/peerj.8391/supp-2 File S2 The expression data of apple *AP2/ERF* genes under normal growth, mannitol and salt treatments

10.7717/peerj.8391/supp-3 File S3 The expression level of the response of apple *AP2/ERF* genes to AAAP infection

10.7717/peerj.8391/supp-4 Table S1 Application of primers and sequences

## Additional Information and Declarations

### DNA Deposition

The following information was supplied regarding the deposition of DNA sequences: The MdERF3-8 sequences are available at GenBank: MG099812, MG099813, MG099814, MG099815, MG099816 and MG099817. The MdERF11 sequence is available at GenBank: MG099820. The MdERF16-19 sequences are available at GenBank: MG099825, MG099826, MG099827 and MG099828. The MdERF22-28 sequences are available at GenBank: MG099831, MG099832, MG099833, MG099834, MG099835, MG099836 and MG099837. The MdERF31-35 sequences are available at GenBank: MG099840, MG099841, MG099842, MG099843 and MG099844. The MdERF39 sequence is available at GenBank: MG099848. The MdAP2D60 sequence is available at GenBank: MG099849. The MdAP2D62-65 sequences are available at GenBank: MG099851, MG099852, MG099853 and MG099854. The MdRAV2 sequence is available at GenBank: MG099860.

### Data Availability

The following information was supplied regarding data availability: The expression level of apple AP2/ERF genes in different tissues is available at NCBI GEO: GSE42873. The expression level of the response of apple AP2/ERF genes to AAAP infection is available at https://www.frontiersin.org/articles/10.3389/fpls.2017.00022/full and in the Supplemental File.
