# Supplementary material for: Isolation, sequencing, and expression analysis of 30 AP2/ERF transcription factors in apple
Source: PeerJ. 2020 Jan 17;8:e8391. doi: 10.7717/peerj.8391 (PMC6970539; doi:10.7717/peerj.8391)
Supplement: File S1 [file peerj-08-8391-s001.docx]

>MdERF3 [Leaf=Malus domestica] AP2 domain class transcription factor

ATTTCTGGACTCGGGTTTTTGAGGGTTTTGGGTTTTTCTTGATACCGAGTTCTGAACTTTTGAGAGAATTTTTGTTTTCT

TTTTCTTGTTTTCGGGTATAGGGGGTTTAGGGGTTTTAAGTTCAAAATGCCGGGGATGAAAACCGTAATCTTGAATCAAC

ATGTGAGCTGGAACAAATTGAAGAAAAAGCAGCCTGCCATGGAAGAGAATGCAGGTTACATGAAGAGAATTCGGATTATA

TACAATGATCCTGATGCCACCGACTATTCGAGTGAAGATGGTGAGGAGTATGATATTGAGGGAAATCTATTTCAGCATTG

TAAGCGCTTTGTTTCGGAGATTTTGGTTGTGGAAACGGAATGTGAATCATCTGAAGGGACCAATGGAGGCAAAATTGGTC

ATAGGATGGGCTTTGGTGACTCTAAGAAATGTAATAGAGTGCTCCCTAAGGGAGTTCGACGAAGAAAATGGGGAAAATAT

GCAGCGGAGATTCAAGATCCATTCCAAAGGATTCGAAAATGGCTTGGCACTTTTAATACAGCAGAGGAGGCTGCCGCTGC

TTATCAGCAAAAGAAGCGTGAGTTTGAAACCATACAGTCACAGCAAAAGATTAAGAGCCGCCGCAAAAACATTCAATTAT

CAGATAAGCATGAGTTTGAGAGTTCGCAATTAATGGAGGGAGCCAAGCATGAGCTTCAGGGCGTTCCTTTATCAGAGGAG

GGTCAGGATTCATCGTTTTGCCATCCGTCACCATCATCTGTGCTTGAAATATCTGGTTCAGCTTCGCTTAGTTTTGAACT

CGAAAAGTTGAAGGAAGAATCTTGTGTGGAAGTTTCTGGGGGAGAAGGCGCTGTGCAGCAACCAGTTTTTGAAGAAGAGG

AATCCATTCCAATGTTACCACCATTGGATGTCGAACAGTTGAATTTGGGATGTGACGGCAATTACATGTATTCTAGCTTG

TTGGACCAGTTCTTTGATGGCATGTGTGATATTGTTGATGATGGTATGACAGACATAGTTGATTATCCCGTTTGCAACGA

TAAAAAAGGCGAGGTTATATGTCTTCCCCCACTCGACTCAGATTTTGGTCAGCAGGATTTCTCTTGGATTGATCAGAAAC

CAGAACAGGTTATGCCCATGAAGTTTTGTAGTTAGGAATTACCAGTAGTTTATCTAGTACATGTTGCACAATAAAAAGCT

CAAGAAATGTTTGAGCAGCGGGTGTTTGAGGTTATCGTTTCCTATGTTCGGAGAGTTCTTGGGTTTTCATTGTGCTTTTC

GGGGCCGTCCCAAGATTTTTCCAACTCTCATGACTTAAGAGTGGTAATATGTGTGAGTAAGCTACTATATATTGAACCTT

TGTTTCCTCCTTCGCATTTTGTATTAAGT

>MdERF4 [Leaf=Malus domestica] AP2 domain class transcription factor

CTGAAATATCCATTCCAATATTTGTTCAAAGGAAGAGCTAGAAGAAGAAATTCTGCAAATTGTTCATTCTTCCAYAAAAC

AAGATGGACCCTTCGTTCTTCCAAAACCCTAATTCTGAATTCTCGTTTGAATCTTCTTCTTCCTTCAACTCCCTAGAATA

TTCTCACGATGATCATCTCTCATGGAACATGTTCGACTTTAAAGATTATCACAACCAATCAGCCTTTCCCCAGCTACCAT

TTAATGAGAACGATTCAGAAGAAATGCTTCTCTATGGAGTTCTTGCTGAGGCGCCAGCAGGAGATCAGAACTCGTCGGAT

TCAATAGTATCCTCCACTCAGTCGAAAGACCATGAAGAGGTGAATTCTTCTAGTGCCACAGCTAACGAAGATCAAGTAGT

TGCTTATAGAGGCGTTCGGCGGCGGCCGTGGGGAAAGTTTGCGGCGGAGATAAGAGATTCGACGCGAAACGGTGTTAGGG

TTTGGTTGGGAACTTTTGACACGGCAGAGGCGGCGGCTTTGGCTTACGACCAAGCAGCACTTTCAATGCGGGGGTCTTCG

GCGGTCCTCAACTTTCCAGCTGACGTGGTTCATCAGTCACTTGTGGAGATGAAGTATGGCTTTGAGGAGGGTTCGTCTCC

GGTTTTGGCCTTAAAGAGAAGGCACTCTATGAAGAGAAAATCATCGATGAACAAAAAGAAGAAATCATTGCATCATAATC

AGCATCAAAAGAATGATGAAGAAGGTGAAGATCAGTTGGAAAAAGTTGTGGTGTTTGAGGATTTAGGCGCAGACTATTTG

GAGGAGCTACTCAGTATATCCGAGAGCTAACTAAAGTGGAATGGTACATCACGTCGTTTGTGTTTTCGATAACCAATAAA

ACTTTTTCAAGCTAGTAGAGCTAGCTGTCCTTAATCACGTTGGTCCATCTGTGGCAAGTGTTAACCATACCATTTTTGGA

CCTTGTATTTTTGTTTACTATTTTTGGGAAAGTATGTGGAGAGATGACGGTCAGTGGTCA

>MdERF5 [Leaf=Malus domestica] AP2 domain class transcription factor

CTCTATACCAAGATTCCGCCTAAGACCAGCCAGCTAGCCACCGAAGCCAGTCCTCACCCATCACCCATTTTTAGGATATT

TACCCTGCTGCTCTGGAAGTACAGATTGCAGTGCGCACACTCTCTCTCTCTCTCTGTCTCTCTGTCTCAACCTTCTACAA

ATTCAAAACCTCACTCACTTCATCTCAGTCGTACTAACTTTTCTACATGCTCTCGCCGTCGATTCTTCATGTTGCTGCTT

CCGACCAAACCCACATCTCCGATTTCATTTCCTCCGCCTCTAAATCCATGACAACCTTCCAACCCACCTCGTCCGCACAC

TCAGCGGTCAAATTTTCAGAGCAAATCACCACCGTGACAAAACCCATGATCCAGGAACCCGATAACCCGATCCGAGTCCC

GCCAAAGCTCGTCCGGATCATCCTCACGGACGCCGACGCCACCGACTCCTCCAGCGACGACGAAGACAGCGGCGTACGCA

GAGTCAAACGACATGTCAGACAGATCAGCCTCGAGCTCTCCTCGCCTTCCTCTTCCTCGCCTTCATCTTCGTCGTCGTTT

TCTTCGGGTCAGTCGAAGCGGGCCAGGAAAGACCCGAAACCCGTAAAGCGAACCGGGTCGAAATCGCCGGCAATGGATGC

ATCGCGCCAGAAAAAGTTCAGAGGCGTACGCCGGCGGCCTTGGGGTCGATGGGCTGCGGAGATCCGAGATCCGACTCGTA

AAAAACGGGTCTGGCTCGGAACCTTCGACACAGCGGAGGAAGCCGCCAGTGTTTACGACAGAGCTGCCATCCTTCTCAAG

GGTCCAGATGCCATCACCAACTTTTCTAACGCCGTTACAACGGAAGAGAAAGTCGTTGCCGATGTGGTGGTCCCAGCTCC

TCCGCAGCGATGCGGGTGCGTATCTCCGCTGTCCAGCGAGGCTCTCTCGTCGCCTACCTCGGTCCTCCGTTACGAGGAGC

AAACGCCGTTTGATGGTTTCATATACGGAGATGTTGACGACTTCGTGTTTGATATCGACTTGCCGTTAAGCTTTCCAGAT

ATTATGTTGTCGAGTAACAACTTTGTGCGCGACGAGTTCGGCGAGTTTAACGTTGACGATTTCTTAGCTGACGTCGTAAG

TTGAACCCAGCAGTACTCTACACGTGTAACTACAAGTAGGCAGGATTATTTTAGTTTGGGACTGTGCTGCAATTTGCAAG

TGAAGTGAGTTTTGTTAAGTGGCGCAAGCAGTACATGGAATACCGAAAATGTCCTCCGGAGTGTGATGTACAATCACTAC

GAGTATAGCCGATTATCTCTAATATATACGGCGGTTT

>MdERF6 [Leaf=Malus domestica] AP2 domain class transcription factor

CTATCTAAATCTCAACCTTCTACACATTCAAATCCTCACTCACTTCAGCTCAGTGGTACTAATTTTTCTACACGCCCTCG

CCGTCGATTCTTGATGTTGCTTCAGACCAATCCCAAATCCCTGATTTCAGTTCTTCCGCCTCAAAATCCATGACAACCTT

CCAACCCATCTCGTCCGTACACTCAACGGTCAAATTTTCAGAGCAAATAACCACCGTGAAAAAACCCATGATCCAGGAAC

CCGATAACCCGACTCGAATCCCGCCAAAGCTCGTCCGGATCATACTTACGGATGCCGACGCCACCGACTCCTCCAGCGAC

GACGAAGACAGAGGCGTGCGCAGAGTCAAACGACATGTTAGACAGATCAGCCTAGAGCACTCCTCGCCTTCCTCTTCCTC

TCCTTCATCTTCGTCGTCGGTTTCTTCGGGTCAGTCGAAGCGGGCGAGGAAAGACTCGCGACCCGGATCGAAGTTACCGG

AAACGACCGCGGATCGCCGGAAAAAGTTTAGAGGCGTCCGGCGGAGGCCTTGGGGTCGCTGGGCCGCGGAGATCCGAGAC

CCGACCCGAAGAAAACGGATCTGGCTCGGGACCTTCGACACGGCGGAGGAAGCCGCCACTGTTTACGACAGAGCTGCCAT

CTTGCTCAAAGGTCCTGACGCCGTCACCAACTTTTCTAACGCCGTTATAAAGGAGGAAACAGTGGTTGGCGACTTGACGG

TCCCAGCTGCGGGGCAGGGATGTGGGTGTGTATCTCCGTCGTCCAGCGAGGCTCTCTCGTCGCCTACTTCGGTCCTCCGT

TACGGTGACCAAACGCCGTTTGATGGTTTAAGTTACGGATACGCTGACGCCTTCAAATTTGATTTCGACTTGCCGTTAAG

CTTGCCGGATATCATGTTTTCAAGTAGCAGCTTTGCGCGCGACGAGTTCGGCGAGTTTGATGTTGACGATTTCTTGGCTG

ACGTCGTATGTTGAATCCAACAGTATTACACACGTGTTGACTCCAGGTTAGGCACGTGGATGTTAGAGGAGTATTTTGTA

ATTTCACTTGCGACAGTGTTGCAAGTTGCAAGTGATGTGTGGCTAGCTAGTTGCAAGTTGCGAGTGAAGTGAGTTTTGTT

>MdERF7 [Leaf=Malus domestica] AP2 domain class transcription factor

TATAGTCTCTCCTTCTCTGTCTTTTTATATCCACCTATCTATCACCACATGTAGCCTCAAATTTCCATCTGTTACCTTCT

CTGCACTTCTCTGAACTCTCAATTTATCGTCCCTTCATTCTTCTCTGTCTCTCTCAGAGTTAGACAAACAAACAAAATGG

TGAAATCGAAAAAGTACAGAGGCGTCAGGCAGCGCCACTGGGGCTCTTGGGTCTCAGAAATCCGCCACCCCTTACTGAAG

AGAAGGGTGTGGCTAGGCACATTTGAGACAGCTGAAGAAGCAGCCCGAGCATACGACGAAGCTGCCGTTCTGATGAGCGG

ACGAAATGCCAAGACCAATTTCCCAACATCTATTACAACTCAGAGTAAGAGTACTCGTGCCACAGTAGGATCATCCGATC

ACCCAAAAACTAGTAGTGGTGACTTGGATTCACCATCCGGACCGAAGGGTTTGTCGGAAATATTGCATGCCAAGCTAAGG

AAATGCAGCAAGATACCGTCCCCATCCATGACCTGCTTGAGGCTCGACAATGAGAGCTCTCACATTGGAGTGTGGCAAAA

GCGGGCAGGTCAACGCTCTGATAACTCCAATTGGGTCATGACTATTCAGCTTGGCAAGAAGAAGAATAATAATAATGTTG

ATACTAATAATGCTGATGATCAGAGTGTTGTACCATCACAGCTAATGTCGAATTCAGATCAGTCGGCTTCGATTGCAACA

TCATCAGAGAGGGCGCCCGAACTCATGGCGGAGATTGATAAAGAAGAGAAAATTGCGTTGCAGATGATAGAAGAGCTGCT

TAACAGAAATTGCCTAAGTCCTGATTTATCATTTGGCATTCATCAAGGGGAGGAAAAAATTTAGTTGTAGTAGTACTACT

CTTATTTAATTACTAGCTATTTGTGTTTGTTTGGTAAAATTAAGTAAGTATAGTGTTATATATACCCATCTAAATAATCT

CTCTACATATATACACATAGGTAATGGGAAAAATAATATATTTAATTATGTAGTCATTAAAAGGGTTAAGAGTAATAATT

GTAACCCATTAGTGTCCAATTAAAACGGTTTGGATATTTATGTGCCAAATTGAGAATAATAGATC

>MdERF8 [Leaf=Malus domestica] AP2 domain class transcription factor

GGATCTAACCTGATACCACTCACATCACAAACTCAAATCTCATATTTTTAATTTCGTTTTTTTTTTTCCATTTTTCTGGG

TAAATTTGAGGGTTTTGGGAGAAGATTTTAGGCATTTTTAGAAATTAGTTCAGAAAATGCCTGAGCCTCGGAGACAGCTG

GTAAATCAGCGGAACAGTATGAGAAAGTCCAAGAGAGAACTATTTCCTGCTCAACAGTCCAAGATTATGAGGAAAGTCCG

TATTATTTGTTACGATCCCGACGCCACTGATGACTCATCTTCAAGCGAGGACGAAGGAGGAAGAGAGAGAAGATTTCCCA

ACAACCCAAAACGCTTCGTTCGTGAAATTACTTTCTGTCAACCCAATGTTGCGCTCCCGCCGCCGAAGGCCGCCGCTGAG

CCGGAGAGCTCAGGTCAAGACAGCAACTACGGTGGAGTTAAAACCCTGAAACCCAGCAACAAGAAGGTGATTTTCACTAA

ATCCCATCGGCAGTCCTCTTCTCCTTACAGAGGAGTGAGACAGCGCAAATGGGGGAAATGGGCAGCTGAGATTCGTGACC

CTTTCAAAGGGGCTCGTATCTGGCTTGGTACTTTCAACACTGCAGAGGACGCTTCCAAGGCCTATGAGGCGAAGCGGCTT

GAAATTGAAGAAATGGTGGCAGCTGCTCCGGCGGCCGTTTCAGCCTCTGATAAAAGCAACACCACCTCGGCTTCAGTTGT

CGTTTCCAACAACTGTTCCTACAACAACGTTGAACCTGTTTCGTCTGAGGATTCTGACTCTGTCGTCTCTCAGAGCTCTC

CTTCTTGTGTGCTTGATTTGGAGACATCTGCTTCCAATAACATTCGGAATGGTGCTGATTTGGAGAAGGAGATTGCCGAT

GAGTCGACAAATCTTGAAGAGCTGCAAATACCCGATTTGGGTTTTCTTGATGAGACATTGGGCCCTCTTCATTTTGATCA

AGAGCTAAACTTGGGACCGGAGCTCGATTCGCTTTACCTGGAGGATTTTGGGCAGTTGTTCGACAATTACAGCAGCATTG

AGGATATACAGATTCCCGGGTTCGAAAGCGATGAGCCGAGCAGTCTACCCGACTGTGACTTTGAGGATCTTGGCAAGGAT

GACATTGCTTGCTGGTTGGATGAACCCCTCAACATAGTATGCCAATAAATTTTGCAGCTTCTCTTTAGTTAAATCTCTGT

TTATGGTTTTGCAGCAATTAGGCGTAATATCATGAACTCTCAAGTAACACATGGAGTTCTCGAGTGAGAGTGGTAATTAT

GTGAGAGTTTAATTATTTTGTTTGTTTAAAGAGAGATTAAGATGGTGATTTGCGGCATAAGCGTTTTAGTGTTAAGTGTG

AATCCTTGGGTTTTCTTGTGCTATCAGAGCCGTCCAAAGGGTTCTCGATCT

>MdERF11 [Leaf=Malus domestica] AP2 domain class transcription factor

CTGAGGCGTTTAGGGCTTATTTGGTTTTTTCCCTTGTCATTTTGTGCTGTGTCAGTTGTGAGCTTGAACCTCAAACTCTC

TTTCCATGGAGCATTGACCATTTTTATCTTCCAAGCTCTGCTGCCCCCTCTTCAGATACCAGCCTCAATTCTTAAAGGGG

GTCCCGGAACAAAAACAAGGTCACTCATCATTATTTTTCCTGTTCAATACAATTTGAACTGCATGCCCAAGTTGGTCAAG

ACCGATTCGAGACGGGATGGACCCAATCCTGTGGCCGAGACTCTTGCGAAGTGGAAAGAGTACAACGACCATTTGGATTC

ATGCAATGATGAGGGTGGACCGTTCCGTAGAGTACCAGCCAAGGGATCAAAGAAGGGATGTATGAAGGGTAAGGGAGGAC

CTGAGAACTCTCGCTGTAACTACAGAGGTGTTAGACAGAGGACATGGGGCAAGTGGGTTGCAGAGATCCGGACACCCAAC

AGGGGAAGTAGGCTCTGGCTAGGCACTTTTCCAACTGCCATTGAAGCTGCCCTTGCTTATGATGAAGCGGCAAGGGCCAT

GTATTGTTCTGCTGCCCGACTTAACTTCCCTAATATCTCAATTTCCACTTTATCAAAGGATTCTTCATCGACAAAAACTC

TGTCCTGCCTTCCCTTGTTAGCATCATCTGCAGGTTCAGAATCTTCAGCGAGATCAGACCACTCGGGGGATTGTGCCGCT

CAGGATGAGGATCAGGTGAATGGTCTTTCATCTAATGTAGAGAATGATGATACGACGAATGTAGATCAAACAAATGAGGA

TCGAGATGATGAATGAAGAGTCGGTCTTTGATCTTCTTGGCCATAATGTACATGTTCAGCATACATACACGGTGATTGTG

GGAGATGAGCAATTTTTTATTTTATCTGTCAAATTACACTTGGGTTGGATGAAGTTTTCATTCGTTCTAATCTGTTGTCT

TTTGATTTTATAAA

>MdERF16 [Leaf=Malus domestica] AP2 domain class transcription factor

TTTCTGCATATTCGACGCCTGAAGGAAGACTTCGGAAACCGACTCAGATAAAAAGGAAGAAGAAAGCAGGCAGATTTCTG

AACTTGCATGGAGTCCGAGGCTTCGGACGGGGAAAGGAAATTGCGGAAGAGGCGCAACGGGTGCGAATCGATAGAGGACA

CACTGACCAAGTGGAAGAACTACAATGAGAGGCTTGATTCTGGGAAAGATGGAGGGAAGAAGACTCGGGCTCCGGCGAAG

GGCTCCAGAAAAGGCTGCATGAGGGGGAAAGGAGGGCCGGAGAACTCAGATTGTGTATTTAGGGGTGTTAGGCAGAGGAC

TTGGGGAAAATGGGTGGCGGAAATTCGAGAACCAATTCGAGCTAGAGCCGGTTCTGTACCTACGAAGAAGAATAGGCGTC

TTTGGCTTGGTACTTTCCCTACAGCCTATGAAGCTGCTCTTGCTTATGATAAAGCTGCTAGAGCAATTTATGGTGCTTTG

GCCCGGCTCAACTTCCCCGACAATGCTGTGGACTCGAAGGACTACTACTCTAATTCTGTGTCATCTAAAACACCATCGTC

GCATGAGTCCTCATTGACATACAATAATGCGGATGCGGCAGGGGGAAGATCAGGATTCTTTGAGGATTGTGTGGCCAAAG

AGCAAAAGCAGGAAACGGATTGTTCTGTATCAGAGGAATTACATGTTTTACGTGCTACCTCAAGAGAACCAAAAGCTGTG

AAATGTGAATACGAAACTGAACGTGAGCTTGTTAAGAACGATGATGTTTTTCAGACCGAGAGCTATGGAAGCTTCGATCA

TAGGGGAGATTACTTGCCTAATGAGCCCCTGGTTGTGAATTTTGATACGGTATTTGATTGTAAGCCTTGTAACGATATGG

ATCCTTTGGAGACACTTTTGAGGTCCAATTATGATTACTTAACTGAGCTTGTAGATGGGGAATGCAATCGGAGAAACAGT

TGCAAGCCTTCAAATGATGTCAAAGTTGAGACACCGGCAATGAGGGAAGCCGCGGAGAAACCATTTCCGGTGATTCTGGA

ATCCGGAAGTCACAACGGGTTAGATGAGAAGTACAACAACATACATGGTGAGCAAATAAACGCGGCCGAAATTCTTGTAA

CCAATTTTGAGCCTTCTGAGGATGTTGAAATGAAGTTATCGATGACGAATCAAGAATTGCAAGGAGGATTTGCAGAAACA

ACGAGATTAGATGGCCATAATTGCAATGGCTTCATACATAGTTATGCTTGTTTAGATGATTTAGATGTGGCATACGGTCC

GAGATATGGCATTAATCCTTGGAATGATATCGGAATGCAGACAGAGCTCGACGACAGACTTGACTACGTGCATAACTGGT

CTGCAGAGGAAACCTATGGCATTGATGCTGCTGAGGACCAACAATGGGGGAGGACGCATAATCTCCCTATTCAGTTGCAG

ACACAACCACATCCCGACATACCTGGAAGCTCGAATCACACGGAGTACGCACATTTAGGCGTGGATATCGATCGACAAAG

TTACGATTCTGGTGCAATGGAAGAGCAGGGGCTGCCTAAATGATTCATGGTTCCCTTACTCTTAATTGAGATTGTTATTT

GGATTAAATAGGAGATTTTAGCTGCATCTTTAACCCAAAAAATAATAAAATCTATATATTCC

>MdERF17 [Leaf=Malus domestica] AP2 domain class transcription factor

CGACGACGGGTTTAGTAAATGGGTTTGCGCGAACAAAACGACGGCGTACGGAGGAGGAAGAAGTCATCGACGAGGGGGCA

CCACCGGTTCGTGGGGGTGCGGCAGAGGCCGTCCGGGAGGTGGGTCGCAGAGATCAAGGACTCTCTGCAGAAAGTGAGGC

TCTGGCTTGGGACTTTTGACACCGCAGAGGACGCGGCTCGTGCCTATGATGAGGCTGCCCGAGCACTCCGCGGTGTCAAC

GCCCGAACCAATTTCGAGCTGCCACAGAGTAATGGGGCCGGAAATGGTGGCGCGGGCGTGGCGGAGAATGTGCCTTTTTC

GTTTGAGGCGGTGTGTGGGAATGAGGTGGAAGCTGAGGGGTTGCTGGGTGCACTTAGAGCTAAGCTGCAGGATGGTAAGG

TGATAAATTGTGTGCTTCCACAACCACCACAAGGAAATAGTGGCACTAAAGCGGAGCCGTCTCCGGTTGATCCACGTGGT

GCGGTGGCGGAATCCGTCAACCTGCCACCACCCCACAAGGACTATCCAATTGTATTAGATAATCAGTGGCAACTTCAGGT

TCACCCAGCACCATCAAACCAAACCATGGTGTGGGCCAACGAGAGCCAAGTAGCATATGAGCAAGTGCATAATTGGTCCA

CGTGGCCGAATAACAGTATGCCGTATGTACCAGAACAGTGCTCTATGGAGCTTCCGTTGCCGGCGGTGGGCACGCAGCTT

CTGTCACAGATTGATGGTAGTGGTGGTGGCGGCGATGGTGGTTGGTGTTCAGATCAGCAGCAGTTTTTGCACTGTGACGG

TAGCAACTGGGGAGGAGCTAATGCCAATTGGGATCCTCCTTTTTATGCTTCCTCTGTGCGGGGTGATGGGATCATATCAT

ATAATTAATCTATGCAATTCTTTTTGTTTTACCATATTAACCTTACAAGCTAGTCTTTGCAGACTAATCAATTCTTTCAA

GTACGTGCCCCAATAAATTATAAACCCTACTCGGGTACGCATTTCCGACCGTCATTCATAAGTAACTCACGAAAGTATGA

GAACTTTTGTAGTTCAGTATGAGAGTTAGCTCTATCAAGGTTTCTCTTATTTGCGTGTTGTAGTAGTCAGTGACTACCTT

AAAGAAGATGTAATCGATATTTGTACATTTATATATGGTTTAGTTAATAATTTTT

>MdERF18 [Leaf=Malus domestica] AP2 domain class transcription factor

ATGGAGGAAAAGTTTCCCAAGATGGAAACCTTCATAGAAAAGCAATTGCCATACTCTTATTTTACAAGAATGGCAACTAG

TAACAATATTGGATCAAATTTGTTCGGTGATCATCCAGCCAGCTGGGGCGGCAGTGGATCAAAATCGCCACTGTCATCGG

AGTCTAGTAGCTCTTCCGCAGAGGATGTTTCTCTTTCAGGTTCCAATTTGATCAACAACACAAGTAGTCCTTTTGTTCCT

CTAAATTTCTTGGAAACATTTCCACAGCTGACTAGAGCTCAAGTTTCTGATCAACCACCTACTTCTCCACCTTCCTTGTC

ATCAAAATCATCGAGATTTCCGAAATTAACTTTGTTTTTGCAGCAACCAAGTCTGTTCGATCCATCATCATCATCATCCC

CATCGTCATCGTCGTCCATGCAAGTTGAAGTTGTAAACTCACTTGGCAAGACCAAAGGGTGTAATGAGTTATCCTCTTTA

TTAACCCCTTCATTTCCTGCTCCCCAAGCAATTGATCAAATTCAGCATCAGCCGGGCATGATTGAGTGGCTTAAAATTAA

TCAAACTATAGCAAACCACTCATCCAAAGGCTCCAATGATTACTGGCTAAGCACGACGAAGACGCAGCCAATGAAGCACA

CTGGAAGATCATCATCAAGATTGCATCAGCAGCAGATCAATCAACAACAAAAGCAGCCTAATAACAATTTGTCCTCCTCA

TCATCATCACAAGGAAAGCTATTTAGAGGAGTTAGGCAGAGGCATTGGGGGAAATGGGTGGCTGAGATTCGATTACCAAG

AAACCGAACAAGGGTTTGGTTGGGGACTTTTGACACCGCTGAGGAGGCTGCCATTGCATATGACACCGCGGCTTATATAT

TGCGCGGCGAACATGCACAATTGAATCTCCCAGGTTTGAAGCATCAACTTAAGGCCAGTGCTTTGAAAGGAACCACAGCT

GCTCTTCTTGAAGCAAAGCTGCAAGCAATATCATCATCACAAGCACATAAGAAGGCCATCAAGGAGTCCTCTTCGTCCGA

ATCAGCATCATCAAACAAGCACTCAGCGGGTGATGGCGGCGATCACAAGAATATCATGATCAATAGTAATTTGAGCCAAA

ACCCTACAAGAAAAGAGTGGGAGTTTGGGTTGCAGAGCAAAGTTGGGTCTCATCACCATCACCACCAGCAGGATTTAGTA

TCAACAGCAGATGTGGATGCTGTTCAATTAAGTAGAATGCCCTCCTTGGACATGGACATGATATGGGATGCTCTTCTGGT

CTCTGATTCCTAA

>MdERF19 [Leaf=Malus domestica] AP2 domain class transcription factor

TTTTGTTCAGCAGAAAAATAACGAAAGCTTTTCTCTCCCTCTCTCTGATCGCTTGCTTTACTTCCAAAACACAAAAACCA

GTCAGTGGATTCAACTTTTTCACCTGAAAAATCCCAACTTTTTTAATTTTTCTGACACCCTTTTCAGCTGCAAGTCCTCC

GTGAAACAGCATAATGTGTGGAGGTGCTATTATTTCCGATTTCATAGCGCCGGCGGCACGGTCCCGGCGGCTGACCGCCG

ACTACCTCTGGCCCGATCTCAAAAAACCCAGTTCGGGAAAGCGGTTCTCGAAGCCTCTGAGGTCCGAAATCGTTGACTTG

GACGACGACTTCGAGGCTGATTTCCAGGAGTTCAAGGACGAGTCCGATGTGGACGAGGACGATGAAATGGTTGATTTCAA

GCCCTCTGCCTTCTCTGCCGGAAAGCCCTCTTCTGCCCGTGGTTCTACTGCTGTGAAATCTGTGGAGTTCAATGGGCAAG

CTGAGAAATCTGCAAAGAGAAAAAGGAAGAACCAGTACAGGGGAATTCGCCAGCGCCCATGGGGAAAGTGGGCTGCAGAG

ATCCGCGACCCAAGGAAAGGGGTTCGGGTTTGGCTTGGAACATTCAACACTGCAGAAGAAGCTGCAAGGGCATATGATGC

CGAGGCACGCAGAATTCGTGGTAAGAAAGCCAAGGTTAACTTCCCTGAAGAAACCCCTCGTGCTTCTGCAAAGCGTTCTG

TGAAGGCAAATTCTCAGAAACTGATACCCAAGACAAACGTGAATGGCACTGAGTCTAATCCGAACCAGAATTTCAATTTT

GTGAACGACTCAAGTCAGGACTATTACAGTGCTCTGGGTTTTCTGGATGAAAAGCCAACATTGAATAACTTTGGGTATAT

GTCTACCTTCCTTGCCAATGGAGATGTTGCACTGAAATCCTCTACTCCATCCGATCCTGCCCCCTTTTATTTGAGTTCCG

ATCAGGGAAGCAACTCATTTGATTGTTCTGACTTCGGCTGGGGAGAACAAGGCTCAAAGACTCCAGAAATCTCATCAGTT

CTTTCTTCTGTGATGGAAGAAACTGATGACTCACTGTTTCTCGAGGATGCTAACCCAACGAAGAAGCTGAAGCCCAACTC

ACAGGATCTGGTGCTTCCTCAGGATAATGCAGGAAAGACACTGTCTGATGAGCTCTCAGCTTTTGAGATGAAGTACTTTC

AGACCCCATATCTTGATGGGAGCTGGGATGCTTCAGTGGACGCCTTCCTTAACGGAGACGCAACTCAGGATGGTGGTAAT

CCGGGGGACCTATGGACCTTTGACGATCTGCCCGCAATTGTTGGAGGAACTTTCTGAGAGATGAACCTTACCCCAGCTTT

CCAGTTTATGTAAATAAAGCTACATGTTAGTGAGTTTTTCAGTCCTCTGTGAGCTTCTACATTGTTTCATTATTGGTCTC

GTGTTCGCTCCCATTTTCTCCAGAAAGAGTTGTCGGTTTCCTTTACTTGTGCTTGCAGGATGTGTTTCGGGTTGGAGTGC

>MdERF22 [Leaf=Malus domestica] AP2 domain class transcription factor

CAAAAGGCTTTCAGACCAAGCAAGCTACCGACAGTTCTTGTAAATGCCTATACTCTTCCCTCATCTCTCTCTAAAATTCC

ATCTCTCAAATTCTCTCTCTCTGGTTATTTCCTTCTAAAAAAACATGGTACAATCAAGGAAGTTCAGAGGAGTCAGGCAG

CGACAGTGGGGCTCTTGGGTGTCAGAAATTCGCCACCCATTACTTAAGAGGAGGGTGTGGCTAGGGACATTTGAGACAGC

AGAGGCAGCAGCAAGAGCATATGACCAAGCAGCAATTTTGATGAACGGACAGAATGCCAAGACCAATTTTCCGATATCAG

ACAACAATTCCGACCAAGAAACGAAACCTGCCACCGACCAAAACACTCCATGGTCTCCGAAAGCACTTTCCGAGCTGCTC

AGCGCCAAGCTCAGGAAATGCTGCAAAGACCCATCTCCCTCCCTCACTTGCCTGAGACTCGACAATGACAATTCCGACAT

TGGGGTCTGGCAGAAGCGCCCTGCTGGATCGCGGGCGAGCTCCAATTGGGTCATGAGGATTGAGCTTGGGAAGAAGAAGA

ATCAGAGTTCTGATCATGATCATCAAGAGGAATCACTGTCATCCTTGACGTCATCGTCAATGGTGGGGTTGACTGAGGGT

GGAATGAGTGAGGCAGATCAGGATGAAGAGGATAAACTTGCAATGCAGATGATAGAGGAGTTGCTGAATTGGAATTATCC

AATACCTTCAGCCACAAGTAATGTTCACGAAGGAAATAATATTTAAAGGGAATTCTATATGCCGGAGTTCTTAACCGTTA

AGTTCTTGATAAAAAACAATAATTGGAATTTAACAGTTAAAAATTTCGAAGTATAGAATATTTCAAAGTACAGTAGAAAT

ATCGATAGATATTACCAACCATCTGAGCTGCTTGAATTCCTAGTGTTATTAGTCCTCGAAGTAAGGAGGCAGTTAAGTGC

TCAATTTGCAGCTACGTACGGTAGTTAATTAAATCATAAAATGCCACCACACTGTTGTTTTGTGATTGGTGATCAACTTG

>MdERF23 [Leaf=Malus domestica] AP2 domain class transcription factor

ATGGCTGCAGCCAAAAAGAGTGGTAAATCCAGGATGGAAACAAGCGTAGATTGGGAGGCTAACAAAGATGGGGAGCTTGA

TTTTTCCTTCGAGAGGCCGCCATGGAAGCCCGGTTTTAGTGAGGCGTCCATGGCGTCGAATAGGCCTCTCAAGAAAGTCA

AGAGCCCTGAAAGGCAAGACCCAATTCAATCTTCACCTTCTTTAGCTCATCAAACACCATCGCCAATCATCGCTCCCTAT

CCATCTTCCTCTTCAAAAATAGTTTTTCCGTTTTCTTTCAACGGGTCTCAACAGCCTATGATGCAATTCCCCCCACAATT

CAACCCCACAAATGTGCCTTTATTGCCCTCACCAATTCAACACCAACAGCAACAAAATCTGCAGCAAATGATATCTTTTG

AATCCCAGCAGCTGCAGCAGCAGCAGATGAGTTATCCGCCCCCAGCGACGTTGACTTGGCAGCAGCACCAGCAACTTCTT

CAGTATTGGAGTGATGCTTTGAATTTGAGTCCAAGAGGGAGGGCGATGATGATGAACAGATTGGGACCAGATGGAAGGCC

AATGTTTCGAGCACCGGCAATGCCGTATAATACTACAAAACTTTACAGGGGAGTGAGGCAACGGCATTGGGGTAAATGGG

TAGCTGAAATTCGCCTTCCTCGAAACAGGACTCGCCTCTGGCTTGGCACCTTTGACACAGCTGAAGATGCCGCCATGGCT

TACGACCGCGAGGCCTTCAAGTTGAGAGGAGAGAATGCCAGGCTCAATTTCCCTGAGCTTTTTCTCAACAAAGACAAATC

AGATGTTTCAGTTTCATCAGCTCCGAGTTCAGCTGCTACTTCGCCTCCAAGGCATGAGGGTTCAAAACCAACCAGGAACC

GCAAGCAACCTCAAAAAATCAAGAGCAGCGCTTCGAATATAGAGGCAATGCCACCGCCACCAATTCCACATACCCAAGAA

GAGAATAGTACTGACAATGATTCAGGACTGGAGTCGAACGAGGCTAAAGCGATTGACGAAGTTGAGGTGAATATCGATGG

TGCTGGAGGAGGGAGTTCACAGCAAAAGGAAATTGTTTGGGGGGATATGGCAGAAGATTGGCTTAATGCAATTCCTGCAG

GTTGGGGTCCAGGTAGTCCTGTGTGGGATGATTTGGACGCCAACAACAATCTTTTGCTGCAATCACATCTCCCTTTTACC

AATCCAAACCAGCAGGACTATAACATTAACATTAATCCTCAACAAGCGATTCAGAATTCAGGTTCGGGTTCGGGATCTGG

TTCCTCTTCTTCCTCTTCACATCCCACGAAGAACCTCTTTTGGAAGGACCAGGATTGAAAAGTTCATTTATTTTAAATTT

GAGCTTTGCACTTCACACTCACACGGTCACTCCTCTAGGTTGTAGAACTAGTTTTAGCCCATTTCGCTCATCATTTCTCA

CTCGTTCCATTTTTCCGGAACCCTGTCAGAAATTGCATTTCAAACTCCTCCCTTATGCATCTTAGACATCAGTTTAGTCG

TCATCCCCCCTCCTACTTTGTCATCACAGCTGGTTGCCGATTTTCAAAAAGCAACCTATGATCCTAGA

>MdERF24 [Leaf=Malus domestica] AP2 domain class transcription factor

ATGGTTTCAGCTCTAGCTCAGGTGATCGGAAATAATAGTGACCAAATTAATAACCCACTTGATCAAGTGCAAGGAATAAG

TTCGTTAATCACCTCACAGTCTAGCCCTACCGAGACTCAATCTCAACCAGTACTACTTCAAGATCAAGGGAATTTGAGGA

GACAACACTATAGAGGAGTGAGGCGGAGACCATGGGGAAAGTGGGCGGCGGAGATTCGTGATCCGATTAAGGCAGCCCGA

GTGTGGCTCGGCACTTTCAACACAGCCGAGGCTGCAGCTCTAGCTTATGATGGAGCTGCTCTCAGGTTCAAAGGAAGCAA

AGCTAAGCTCAACTTCCCCGAAAGAGTAGTTCAAGGGAGCTCTGAGTCAGGTTGTCTCACAATAACTACTCAATTACAAC

ACAGCTTGAACAATATTCCTCCTGAGGCTAATATTTCTCGACCAACATATTCCAATGTTTTCGATTATGCACGATATAAT

AATGTCACATCTACATCGTCGTCCTCCATGCCATCTCAACAAGCCGCAGAGCTCCGAAGCTTTTCGATGCAGTTTGGTTC

TTCTTCAAGTTCGAGTTCGGGTCCTCCTTATAAGTATAGGAAAGACTTTGACAGAAGTCACTCAAGATGATGAATAGTGA

ACTAATCACTAGGGTGAACGCTTGGAGAGACTGAACGATTAAGGAGAGTAAATGGCTCATATAATTTTGATGCATGTGTG

TTTTTCTTTCGTTTTTTTATTTTTGTTTAATTTCTTCTGGACGAAAACAACTTTCAGTTTTTATCACAGTTTACTTCAGT

TTGTATTTGATGAGGACTTATTTGACAAGAAGCTATATAAATTTCCCATGCATGTGTGTGCAACCTTACATA

>MdERF25 [Leaf=Malus domestica] AP2 domain class transcription factor

ATGAGAAGGCAACTTGGGGAGGCAGAACTGACTAAGGAAGCTATGGCTTTAGCCTCCTCACAACAAAATGAGCTCCCATT

GAATGAGAATGACTCACAAGACATGGTCATATACCATGTCCTAAATGAAGCCACTTCTCTCACCCCCTCACTATTGCCAC

AGAGGCACCACATCCATCGTCAACCAAATCGCCTGGAACCCACCAAGAACGTCGGAAAAAAGCACTATAGAGGCGTGAGG

AGGCGTCCGTGGGGCAAGTACGCAGCTGAAATCCGTGACTCTGCAAGACAAGGTGCGCGTGTATGGCTAGGAACATTTAA

CACAGCCGAAGAAGCTGCCTTGGCATATGATCGAGCAGCTTTTCGAATGCGCGGTACTAAGGCCATGCTTAATTTCCCAG

CTGAAATTGTGGCTGCATCATCTCCACCAACATCATCAGTTCATAGATTCAGGCCAAGTTTTAATATACCTTGCAGCTTA

AATAGTAAAAACAGTACTACTACTTCAGACTCAAGTGGAAGTTCAAGTATGCTTTCAGTTGGGACGCCAAGATCAGAATC

TGAGAGTAAATTAAGTGCGGTGGAGGTACAAAATATGGAGGGTCAGATTTTCTAGAAATGTTCTTTTTATTTTTATTTTT

TTAGTTATAAACTTCGGGTTACTAAACATTAGGACATTAAGTTTCATTTTATAGAAAGCAGAGTTATTAGATGTGTGTGG

GAAAGTTTGTAATTTTTCTTTCATTGTCTAGTTATTCTCTTTTGATTTGCCAACACAAACGATAACAATATAACTCACAG

CCAAAGAAAGAAAAAAGGGTATACAAATGTACTGCAAGAAGTGAATTTTATTGGTTTTGTAATATATATGATTGAATGAG

TTGGAGCTTATTTTGTATAGA

>MdERF26 [Leaf=Malus domestica] AP2 domain class transcription factor

ATGGCTAGATCCCAGCAGCGGTATCGAGGCGTCCGACAGAGGCATTGGGGCTCTTGGGTCTCCGAGATTCGCCACCCTCT

ACTGAAGACACGAATTTGGCTAGGCACATTCGAAACTGCGGAGGACGCTGCACGAGCATACGATGAAGCAGCAAGGCTCA

TGTGTGGCCTAAAAGCGCGCACAAATTTCCCATACAATCCTAACGAGCCCATGTCGCATTCATCCACGCTTCTCTCAGCA

GCTTTGATGGCAAAGCTGCACAAATGCAACATGGCCTCTCTCCAAATGGCCAAAACTACCAAACCTTCAGTGCCAAAGGA

GGAGCCCCAAGAACAAGTACACATGCCGCTCCCTTCTACCACCTGGATTACCGGAAAAGGGGGCGAGAAGGTCAGGGATT

ATAGCGGAGCAGAACAATGGGTAGATCATGGGAGCTGGGTTGGTGGTGATCTGCAGGGGGTTCCTCATGATCATCATCAT

CATCAGCAGGAGTTTATTAAGCCTCTTGAGGATGATCATATTGAGCAGATGATTGAGGAGCTTCTTGATTATGGCTACAT

TGAGATTTGTCCTGGTGTTTCTGCATAGGCAACGATGAACTCAATTGCAGCTAGAGCTAGCATTTTAGAATCTTGTGTGG

AAATGCAAAATTTTTTGTATTAGCTTATATAGCCTTATATGTAATAAAATTAGTTAGATGAGTTTAAATGAATGGTTATT

GAATAA

>MdERF27 [Leaf=Malus domestica] AP2 domain class transcription factor

ATGAAGTCTTTGGATGCGGAGTCGTGTATTTTGTGTCCAATCAAATACACGGAGCACAGAAGCAGGACAAGGAAAGTGAC

CAAACCGCGAAAGAAGTCGACAGCCGAGTTCAACTCGTCAGTCATCCCGAGAATCGTACGAATATCGGTGACCGATCCCG

ACGCCACGGATTCCTCCAGCGACGAAGGGGAGGAGTTTTTCGGGCGGCAGAGAGTGAAGCACTATATTAACGAAGTCAAC

ATCGAGAAGGACTTCAGGAACAGCCTTGTCATCAAGAACAACGCCCGGAAGCGGCCGGCTGCTGAGGTTCCGGCCAACCG

GCGGCCAATGAAGAACGCAGCTTCGCCGACCAACGGCGGCAAGAAGTACCGCGGCGTAAGGCAACGGCCTTGGGGGAAGT

GGGCGGCTGAGATTAGAGATCCTGCCAGACGTCAACGCCTGTGGCTGGGGACCTTCGACACGGCGGAGGAAGCCGCCATG

GTGTACGATAATGCCGCTATTAAGCTCCGCGGTCCCGACGCTTTGACTAATTTCGTGGCTCCGCCGCCGAAAGAGGAGGA

AAAGCCAGAGCCGCTGGTTAACGAGCCTGTCGAGGTGGAGACTAACGGAACATCGTCGGTTTCCGGTTACGATTCCGGGT

CCGAGTCTCACAATCTGGCCTCTCCGACCTCCGTCCTCCATTTCAGAAGCCAACCCATCATTGAAGAAGTTGACGAGGCT

CAAAGATTCCAGATGTTCGACCGAGTCGATGAGTATGAAGACGAAACCGGTACGAACATTTTGTCCGACGATTTGTCCGA

TTTCGCTAACTATTTGCCGCTGGACTTGCCTTACTTCGACGACGCATTCAGTTTCTCAGCTTCGGAGTCGCCGCTGTTCG

ACTCGCCTCTGTTTTTCGACGACGACTCCGCCGCAGCAGCCGATACTCTTCCGGATTGCTCTGTGAAGGACGATTTCAGC

GAGTTGTTCCGGGACACGTTCGACGCGTCGCTGTCGCATACGGCGTCGTCTATGTGCCAAGGGGATGACTACTTTCAAGA

CATTTTGTTTGGTTCCGACCCGCTGGTGGTCCTCTGA

>MdERF28 [Leaf=Malus domestica] AP2 domain class transcription factor

CTTAGGAGGAAGAACTAGCTAGGAAGCTATGGCTTTACACTTCTCACAACAAAATGAACTCCCATTGAATGAGAATGATT

CACAAGACATCGTCATATACCAAGTCCTAAATGAACCCGTGTCTCTCATCCCCTCATTGTTGCCACAAAGATACCCAATC

AATCGTCAACCAAATCGCCTCGAACCCACAAAGAACATCGGAAAAAAGCACTATAGAGGCGTGAGGAGGCGTCCGTGGGG

CAAGTACGCAGCTGAAATCCGTGACTCTGCACGACAAGGTGCGCGTATGTGGCTAGGAACATTTAACACGGCTGAAGAAG

CTGCCTTGGCATATGACCGAGCAGCTTTTCGAATGCGCGGTACTAAGGCCATGCTTAATTTCCCAGCTGAAATTGTGGCT

GCATCTTCTTCACCAACATCATCAGTTCACAAAGTCGGGCCAAGTTTTGGTGTAACTTGCAGCTTAAATGGTAAAAACAG

TACTACTAATTCAGACTCAAGTGGAAGTTGTAGTATGCTTTCAGTTGGGACACCAAGATCGGAATCTGAGACTGAATTAA

GTGCCGTGGAGGTACCAAATATGCAGGGTCAGATCATCTAG

>MdERF31 [Leaf=Malus domestica] AP2 domain class transcription factor

ACTCCGCCTCCATTTTTCTCTCTCCGTACGGTCCACCCAATGTCTACTGTAGACGCGCTCCACCTCCGCGACACCGCACT

CTCCCTTGCCGTCGAGCCGCCTTCGCCTCCGCCTGGTATACCTCGACCTCAGCCAAAGCACGGTGCGCCCGCCAAAGCCG

GCGCAGGCAAGGATCCGCATTTTAGAGGCGTGCGGAAGCGGCCGTGGGGGCGGTTCGCCGCTGAGATTCGCGATCCCTGG

AAGAAGACTCGGAAGTGGCTCGGCACCTTCGACACCGCCGAGGAGGCGGCGCGTGCATACGACGAGGCCGCGCTTAGCCT

CCGCGGTCCCAAGGCCAAGACCAACTTCGGAAATCTCGCTCTTGTTCCGCCTCACTCGCTTCTCCTCGGCGGCGGCTCTG

AGATGTTCTGGCCTCCGCCGCCGATGTACTTTATGTCTGGAGCTCCGGCGGTGGCGCCGATTAGGTCGGAGTATACAGGG

TACAAGCTTGAGAAGGTGGACGCCGTGCGGGGCCAGGAGGAGAAGAAGATGAGGAAGAACAAGAAACCGTTCTTGTTCGA

TCTGAATCTACCGGCGCCGCTGTTCTGA

>MdERF32 [Leaf=Malus domestica] AP2 domain class transcription factor

ATGAATTACTCGACTTTTGATTCCCCAAACTCCAATTTGTCACCAGAATCTTCATTTGGATCATCATTTTCTTGGGATGA

TGGTCTTAAATTTGGCAACAATTCACTTCCTTTCAATGAAAATGATTCAGAAGAAATGCTTCTTTACGGTCTAATTTCGG

AGGCCACCCAAGAAATAACGTCAAGTTTTTTTGTTTCTGCAAACCCAATTAAGGAAGAGGAGGTGAGCTCCGCATGCGAA

GAAGAAAACCTGAAAAAGGAAAAGTCCTATAGAGGGGTTCGGAGGCGGCCATGGGGCAAGTTTGCTGCAGAAATAAGAGA

CTCCACTAGACATGGCATAAGGGTGTGGCTAGGCACATTTTACAGTGCCGAAGCCGCAGCTCTTGCCTATGATCAAGCCG

CCTTTTCCATGAGAGGCTCTACGGCAATTCTCAATTTCCCAGTAGAGAGAGTCCGCGAATCGCTTAGGGAGATCAACTAT

GGTACCTTGGATTTGGAAGGATGTCCGCCGGTGATGGCGCTTAAGCGAAAGCACTCGATGAGAAGGAAAACTGGGTTCAA

GAAAAGTCAAGTTGACAGAGATGTGATGATAGAGAATGTGGTGGTGTTTGAAGATCTGGGAACAGATTACTTGGAAGAAT

TGTTAAATTTCACCGAGAAAACAAGTACCAGCGCCACTCCTAATTGGTGAACCAAAACAGCAAATTCATCTTTATTGAAT

TCTTCTTTTGTTTGTTTGTTCTTTTTTCTGTAATCAATCTCTTTGTAATTGTATGAACCGTGACTCCAATCTTTTGTTTT

TGCTGTAAAGTTCGTAGAAAGATAAAGGTGTTACGCATTAATGA

>MdERF33 [Leaf=Malus domestica] AP2 domain class transcription factor

CTCTCTCTCTCTCTCTCTCCCTTCCTCTAATAAAACCCCTCCCTTCACTTGCTCTTCTTTCTCAATTCCCCTTCTTCCTT

TCCCGTTTTCCTCACTCACTCACACACGCAACAACTACGTACACTCTCCAACTCTCTCTCTCTCTCTCCAGCATTACCCT

ATAAATAATATAACTCGGTCACACTGCCTGAAGCTCACAAAAGCCAACTCCTTTCCACTTCCAGAACGCATTCTATTTAT

AGTAAACCAGAGAAAGAAACGAAAGCTCGATACTTTTGCCTTGGTTTCTTTTTTAAGCATTTCACTGAGTCCACTACCCA

TCAACAATGGCTGACCCAACTCACTCGGAGACCGAGTCGAGCTTCAACTCCTCCTCACACTCCCCTCCTTCCCCGTCCTC

ATCTGGGTTTTCCCACTCCATTCCGAACCCCGGTCAAAATCCACTTCGCGAAATGTCCAACTCGCCAGACCCGGATCAGA

AGCGGGCCAGAGAAACCAGCAGCAAGCACCCGGTTTACAGAGGGGTCCGAATGCGGACATGGGGCAAGTGGGTGTCAGAA

ATCCGCGAGCCCAGGAAGAAGAGTCGGATCTGGCTCGGTACATTCTCGACACCCGAAATGGCCGCGCGTGCCCACGACGT

GGCAGCGCTGACCATCAAAGGCAACTCCGCGATCCTCAACTTCCCCGGACTCGTCGGGTTGCTGCCTCGACCCGACTCGA

ACTCTCCCCGGGACATACAAGCCGCGGCTGCCAAAGCCGCTTCCATGGAAACTCTAAACACGCCGCCTCCTCCACCGTCG

CAGTCGTCTTCGTCGTCGTCGTCAGCATTGTCACAGTCCTCACCATCATCCTCAACGGTGGGTGCGACAAGTACGAGCGA

GGACGCAGGAACGCCGGAGGAGCTGGAGGAGATAGTGGAGCTGCCAAGCCTGGGAACGAGCTACGAGTCGGCCGAGTCGG

GGAGCGAGCTCGTGTTTGCTGATTCGGTGGAGGGGTGGCTGTATCCTCCGCCGTGGTGGCACAACAGTTATTTCGAGGAA

GATTATGGGTATAATAGTCTTATCAACAGTTTTATTAGGGATGATCAAATGTTAATGCCTGAACCACCAGCTGCCGAGTG

TGTTGAAGTTGAGGCCTTATTGTGGCATCATTAAATATGAACCGCTGGATCTTACTTTCTTGTTAGTTGAGTTAATTTAG

TGCCCTTTTTAATTTTCATTTTATTAGGATATTTT

>MdERF34 [Leaf=Malus domestica] AP2 domain class transcription factor

ATACAACCTTGGTGGCCAAGTAGCATGCAAAAAACCCCCTCCCACCTTTCTCTCTCTGTCTCTCTCTCTGTCTCTCTCTC

TCTGTCTTCCAAAAAGCAGAGCACCCCTTTCGCACTCTCAGACCGACATCAACAATTGAAGGAGAACAATTGAAGATGGA

GGGCGGCGGCGGAGAGGCTGCGAATAGGAAGAGAGCTGGGGTGGACAACGACAAGCCGTACAAGGGGATACGGATGAGAA

AGTGGGGCAAGTGGGTGGCCGAGATTCGTGAACCCAACAAGCGCTCTAGAATCTGGCTTGGTTCCTACTCCACTCCTGTC

GCCGCCGCTCGGGCGTACGACACGGCGGTTTTTTACTTGCGGGGTCCCTCCGCGCGGCTCAATTTTCCAGAGGCTTTGCC

GGTCGAAGGTGGCTGCGGGTGCGGTGACATGTCGGCAGCTTTGATACGCAAGCGGGCCACCGAGGTCGGCGCCCGGGTAG

ACGCGGTCGAAACGGCTCTGCGACACCACCGCGGTTCGGCGGCAGGGAACGGAGACGGCGATGAGATTAAACCCTGCTTC

GCCGGATGCGTGGATCGGGTCGACCTGAACAAGATGCCCGACCCGGAGAATTCGGAAGGTGAGTGGAATTAGAGAGAGGT

TGTCTTAATCTCTTGCGGTTTTAAAAATTGAGGGGTGGCCTTTGCTAACGTGCGTAGGGGAGGTATGTGACTGCTGAAGC

AATGATGAAGCTTTCGTCCTGGTCCTGGAAATTAGAATTTCGCCTACTGTATCTCTTTTTTTTACCCTTTTTTTTTCAGA

AAATTTTGCTTTGGATGGAGAAATTTTGGCCATATATAAAAACGTGATGTAAGATGTAAATTATGCTACTAATTAGCTTA

ATTTCCCCCTTTTTTCTGTATTTATTCCTTTTAATTTTGGGTAATTAACATTTGCTCGTATAAGGAAATGTTGAGTCTTG

TAGTTCAAAAAAAAAAAA

>MdERF35 [Leaf=Malus domestica] AP2 domain class transcription factor

GTCATGGCGGAGCAAGTCAACAAAGAGAGTGCTCCTCAAGAAAACCTCCGCCTTCTCCGTCGCTTCCTCCTCCACGACGA

CAATGACAAATTCCGATTCTTTAACCGACAAAGACACTCAAAATTGGACATCCGAAAGAGGGCAGGAGCCAAACATTTCG

AAAGAGCAAAGCCGCAATAAGAAGAAGAAGAGGCAGAGGAATAATGATCACAGCTACGGCGGCAAGCACCCGAACTACCG

CGGCGTGTGGTTGCGCCGGAGGACCAAATGGGTGTCGGAAATCAGCGATAAGGGGAAGAAATTCAGAATATGGTTAGGGA

CATACCCAAAAGCTGAAATGGCAGCTAGAGCTTACGATGCGGCAGCTCTAGCCCTCCAAGGTGCTTCTGCTTCTCTCAAT

TTCCCTGAATTGGCAAAATCACTTCCCAAACCAGAATCCCTGTCTCCATCGAGCATCCAGGCTGCCGCTGCTAAGGCAGC

AGCAGAAGCAGAAGCAGAAACAGCTTTTGGGAAAAAGCAGATTCTATGGGAAGCAGTGACTGAAGAAGCAAAAGCCAGCA

GCATAGATCAAGACCCAACTAGTACTATTGCTTCAACTGATTGTGATGCTGAGAAATTGGTTTCCAATGCAGCTGAATCC

TTCAAAATCTCAATCGAATACATTCCCATAAATTCAGAGGACTGTACATCCGAAAAATGGAGAGAGCCAAACAATGCGGA

AGAGCAAAGCATCAAAAAGAAGAAGAAGAGGCAGAGGACTAGTACTTGTACTAGTGATGATCATCACTGTGAGGGCAGCA

ACCACCAGAAGTACCGTGGTGTCAGATGGCGCAAAAATGGCAAATGGGGGTCGGTAATCAGCCAGAAAGGGAAAAAATTA

TGGCTAGGGTCATTCCCAACAGCTGAAATGGCAGCTCGAGCTTATGATGCAGCAGCTCTAGCCCTCAAAGGTGCTTCTGC

TGATCTCAATTTCCCTGAATTAGCGCAAGCACTTCCCCAATCAGATTCCCCGTCTCCAATGGACATTCAGGCTGCAGCTT

CTGCTTCTGAGGAAATGCAGATTGTATGTGAAGCAGTGACTGAAGAAGCAGATCCCAGGGGAAAAGCTAATCTTCCCATG

TCACTGAATTCAAATAATGCTCAAGAATCAACTAGTACTTGTGCTTCAACTGCAGAAGGATGTGCACACCAAGTGAAGAA

CTTGGAGTTGAATTATCTTGATATGGACAAAGCTTTTGAATCCATCTTCCCTTCTTGGTTGTCCAACTCACCTGAGAGAG

CCTGACATCTCAGCATCATCGCCTCGGCCGCCACTAGAAGATGGTCCATTCTCCTTGTTTACCGAAAAAGATCCACCGGA

CCACTACCCAGTAAAGTTTAGATTTTAGGATTTGTTTCTCGTCTTTGAGGATGTATGTATATAAGCTTTGGATGA

>MdERF39 [Leaf=Malus domestica] AP2 domain class transcription factor

TAATTAAACCCCTTCATTAATGGAACCCCAGGCTGCAGACTGCCGCGTCTCTCCGGCAAGATACAAAGGAGTTCGCAAGC

GAAAATGGGGCAAATGGGTGTCCGAAATCCGCGAACCAGGAAAGAAAACCAGAATATGGCTAGGAAGCTACGAGGCGCCA

GAAATGGCAGCTGCGGCCTATGATGCAGCGGCATTGCACCTCAAAGGGTGTGGGGCGGCGCTTAATTTTCCTGAAATGGC

TGATAGTCTTCCGAGGCCGGCCAGTTCAAGCGCAATGGACGTGCAACTAGCGGCTCAAGAGGCTGCATTGAGGGTTAGAC

GACAGCAGCTGCCAATGGAGGCATTTCCAAAGGAGGAGGCGGGTGACTCGTCGAGGGGTTTGAGCTCAGCGCCAGTGACG

GTGGGACTGTCTCCGAGTCAAATCCAGGCGATTAATGAGTCGCCGTTGGACTCGCCAAAGATGTGGATGCAGTACATGTG

CAGGGCTCATGGCCATGAGGCTCCGTCGTCGTTAGGGGGAGACCTGTGGGACACTGATAATTCCAGTCCAAACAAGTACT

ATGAAGGTGATGTCGAGTCAATGGACTACGAAGATATGCAGCATTGGTCCATTTGGGATCCCTAGTAATACTTAATTAGA

TTATTTATTATAAAATTGAACACTAATAGTGTAGCCAAATATAGAACAAAAATAAATAAATAATACAATATACTAAGTGT

TTGCTGCCTTGGTATCTTTGCCCT

>MdAP2D60 [Leaf=Malus domestica] AP2 domain class transcription factor

AAAAGGAAGAGAGAGAAAAAGCAAGAGGAAGATGAAGTCCATGAATGATCATAACAATAACAATGGTAGCAGTAACAGTA

ACAATAACAACTGGTTTGGTTTTTCACTCTTACCCCACATGAAAATGGAGGATGCTTCTTCTTCTTCCTGCTCAGCTGCC

CTCCCAAGCGGCAACAGCAGCTTCTACAACCTCAGCAGCTCTGGGGTCTGCTGCTATGGAGTTGGAGATAATGGTAATTT

TCACTACTCTCCTCTCTCTGTAATGCCTCTCAAGTCAGATGGCTCTCTCTGCATCATGGAAGCTCTCAGCAGGTCACAAA

CAGAAGGAATAATGCCCAACACATCCCCAAAACTGGAGGACTTTCTAGGAGGAGCAGCTCATGATTATGTGAGTGAGGAG

AGAGAAGCTATGGCTCTGAGCTTAGACAGCTACTACAATGCAGCGGCAGAGCAGCAGCAGCAGAATCATCACATTCCTGT

CCATTCCAATTCCTACTACTCTGGAATCCCAATCCAAGGAATCTACCATACCCAATTGGAGGAAGAACATTCTAAGAATA

CCCAAATGACTCAAATGCCAGAGGACTTAAAAACCTGCTGGGTTTCCAGGCAGTACTCTGCACACCCAGCTCTGGAGCAC

CACCACATGAACAATGTAACCATGGTTGTTGAGAATGGCGGTTCTGGGTCCGTTAATGGAGGCATGAACCGCGGCGATTT

GCAGTCGCTGACACTGTCCATGAGCCCTGGATCACAGACAAGCTGTGTGACAGCTCCAAAGCAGATCTCTCCCACTCAGA

CAGAATGTGCAGTGGCAGCCATTGAAACAAAGAAGAGAGGCTGCGGAAAGCTTGGCCAGAAGCAGCCTGTTCACAGGAAG

TCCATTGACACATTTGGGCAGAGAACCTCACAGTTTAGAGGTGTCACAAGGCATAGATGGACTGGAAGATATGAGGCACA

TCTGTGGGACAACAGCTGTAAGAAGGAAGGGCAAACCAGAAAAGGAAGGCAAGTTTATCTTGGAGGTTATGATATGGAGG

AGAAAGCTGCTAGAGCATATGATCTTGCTGCTCTTAAGTACTGGGGTTCTGCAACTCATATAAACTTTCCGTTGGACAAT

TACACTGCACAACTTGAAGAGATGAAGAATATGAGCCGGCAGGAATATGTTGCGCATCTGAGAAGGAAAAGCAGTGGATT

TTCGAGAGGGGCTTCAATGTACCGAGGCGTGACAAGACATCACCAGCATGGGAGATGGCAAGCTAGGATTGGCAGGGTTG

CTGGAAACAAGGATCTTTATCTTGGAACTTTTGGCACCCAAGAGGAAGCTGCAGAAGCTTATGACATAGCAGCAATCAAG

TTCCGTGGCGCAAATGCGGTCACCAACTTTGACATTACCAAGTATGATGTCGAAAAAATCATGTCCAGCAATACCCTACT

TGCCGGAGAATTTGCTAGGCGCACCAAGGTGATCGAACCTAACATCCTGGCCATTGAGCATAACCCACCAACACAGAACA

TTGTTGAAGCCACTCAAACCGAAATCAACGATGGGAATAGCCTAGACTGGAAGATGGCATTGTACCAATCTGCTGCACAG

CAACAAGCAAACAGTTGTGCTCAAACAGTTGATCAGAAATCAATCGGATCAGGGAGCTATAGAAACCCTTCCTTCTCAAC

GGCATTGGAGGATTATATTGGAGTTGAATCAGTGCACTCAAGTCAGACATTGATGGATGAATCAGCTAGGGTTGGGGCTC

ACTTTTCGAATCCATCGTCGTTGGTGACAAGTTTGGGCAGCTCCAGAGAAGGAAGCCCTGATAAATCTGGCTCCAACATG

CTGTTTGCAAAACCTCCATTGGCATCAAAGTTCATCAGTCCAACTGCTGCTGCAGCTGTTAGCTCCTGGTTCCCATCAGC

ACAGCTGAGGCCTTCTGCTGTCTCCATGTCTCAGTTGCCTCTCTTTGCTGCCTGGAATGAGACCTAGAAAGCTCTGATGT

GTTCTGATTGAACAAGGAAAAATGGGAAGAAATTAGTTATGTTTAGGGAGGGAAAAAGATAAAGGTTGATTTATGTTTAG

GGTAAGTAATTAGTGGAGGAAATTCTGGATTAATCAAAAAGACAATTGTGGGCCTTGGGGGTTCCACTTTTCTGTCTGTA

ACCGAAGTGTTAGAAACGTTTTTTCAAACCAAACCTTTTGGCTCA

>MdAP2D62 [Leaf=Malus domestica] AP2 domain class transcription factor

ATGGATTCTTCTCCTCAGAACTGGCTCCTCGGCTTCTCTCTTTCCAACCACTCTCATCATCATCACCCTTCTCCCGATCT

CTCCCTCTTCGAGGCCTTCACCTCCAATTCACCCACCCACTCTGCAGCTACCGTCGCCGGTCATCATCATCAAGGTGCAA

GTCCAACTTCAGCAACCGACCTCTCCATCTTCAGTTCCGGAATTAACGGACCAAAGCTCGAGGATTTTCTCGGAGGTTGC

ACCCCCGCAGCCAACACCGTCACCCCGACCTCACTCCCTCAGTTTGCTACTGCCGATCATCATCATCAAATCACTCCTCT

CGCTTTGTCCCAAAACGAGATTTACGACTCCGAACTCAAAACCATAGCCGCTAGTTTCCTCCGTGGTTTCTCCACCACCG

CTACCACCACCACCACCGCTGCTACTTTACAAACGACCAAACTACAGAACCACCACCCTCTAGCCTCTTCCGATCCCACC

CCCAAAAAACCCGCCGATACCTTCGGCCAACGCACATCCATTTACCGCGGAGTCACACGGCATAGGTGGACCGGAAGATA

TGAAGCGCATCTGTGGGATAACAGTTGCAGAAGAGAAGGACAAAGTAGGAAGGGCAGACAAGTTTATTTGGGTGGATATG

ATAAAGAAGAAAAGGCAGCAAGAGCCTACGATCTGGCAGCTCTCAAGTACTGGGGTCCGACCACCACTACAAACTTTCCG

GTTATTAACTACGAGAAAGAATTGTCGGAGATGAAGAACATGACTAGGCAAGAATTTGTTGCTTCTCTTCGGAGGAAAAG

TAGTGGATTTTCTAGAGGAGCTTCTATTTACAGAGGGGTCACAAGGCATCATCAACATGGTAGATGGCAGGCAAGAATAG

GAAGGGTTGCAGGCAACAAAGATCTCTACCTTGGAACTTTCAGCACCCAAGAAGAAGCTGCTGAGGCCTATGACATTGCG

GCAATCAAGTTCCGAGGCCTAAATGCTGTGACCAACTTCGACATGAGCCGCTACGATGTGAAGAGCATTGCCAGCAGCAA

CCTTCCCATAGGAGGAATGTCCGGAAAATCCAAAAACTCATCAGACTCCGACAGTAAGAGCATCGAAGGTAACCGTTCAG

CGGATGATCGAGATCTCTCCTCTGCATCCTCCGTGACCTTTGCATCTCAACAACCTAGTTCCTCCACACTAAGCTTTGCA

ATCCCCCTCAAACAAGACCCATCATCAGATTACTGGTCCAACATTTTCGGATATAACCCTTCTCAAAACAATACCAAGAA

CCCTACTACTGTTTCAGTTGCACCATCATCATCATTGTTCCAGTCACGTGCCATTGGGTCTTATGGTAACAATTCCTCAC

CAATACCATTCAACATGGACTTCTCTTCAACTTTTAGCACTTCTGCTTCTGAAACCAACAATGGGTACTTTGGTAACTTC

AATATAGATGGTCAGCAACACCAAGAACAGTTGCAACACCACCAACATGAACAAAGTACTATTACTGGTAGTGGTTCAAT

CCCATTTGCTACACCCATTGCTTCAAATAGCAACAATGGTTATGAAACTTCTTCTGGTTATGGAAGTTGGATTGCGCCAA

GTATTCACACATTTCAGACTCATGCAAAGACCAATCTCTTTCAGACACCAATTTTTGGGATGGAATAATTTGGCTGATAT

GTGTTAGGGGTGAATGTGAGGGTGAGAGATTTATA

>MdAP2D63 [Leaf=Malus domestica] AP2 domain class transcription factor

ATGTTGGATCTTAACGTTAATTTCATCACCATAACTGAGACAAAATCAATGGAGGTTGAAGATTCCGGGACCTCCAACTC

CTCCGTCGTCAATGCCGAAGAAGCTCTGACTCCGAGCAATGCTGGAGACGAAGACTCCACCAACAACACCACTTCCTCTT

TCATGTTTGATATCCTGAAGAGAGAGAAAGGTGGTCTCTGCAATTATGGGGCGGGAGACCAAACGCAATCTATGCAGTTC

GTGACGAGGTCGCTTTTTCCGGTGACCGGAGACGGCGGAGGAGGCAAGGAGGGTGATGGGTACGGGTTGGGATTGTCGTC

ATCGTCCTCGTCAGCGGCAAGGCCTCAGGGGCTGAATTTGTCGTTTGCCGAGTCGGGAGAGCAGACTCAGGCCGAGCTGA

GAGTTGTACAGCAGAAAAAACAGCCGCCCAGGAAGAGCCGCCGCGGGCCAAGATCCCGGAGCTCTGAGTACCGCGGCGTC

ACGTTTTACCGCAGGACAGGGCGCTGGGAGTCGCATATATGGGATTGTGGGAAGCAGGTTTATTTAGGTGGATTTGACAC

TGCTCATTCTGCAGCTAGAGCATATGATCGAGCTGCAATCAAGTTTCGCGGAGTTGATGCCGATATCAATTTTAACCTAG

GAGATTATGAGGAAGATATGAAACTGTTGGGGGATCTGAATAAAGAAGAATTTGTGCACGCGCTTCGTCGCCAAAGCACA

GGAGCCTCACGTGGGAACTCAAAATACAGAGGCGTAGCAGCAGCTGCTCTGCCTAAATGTGGTGCCAGATGGGAAGATCT

AATGGGACAAGTTCCTGGGAATAATGTCTTTGAAAAGAAGGCCGTCAAATGCAGGACCGGAAGAGAAGCAGTTACAAACT

GTGACCCCAATGTCTACGAAGGGGAGATAGTTTTACATGCTAGCATTGAAGGCAGTAGTCACAACCTTGATCTGAACCTA

GGGATTTCTCAACCTTGTTCTACTGGTAAAAAAGGGAATGGAAAATTTGGGGATTTTCAATTTCCTAAAGAGAAGCCAAT

GGTTAATGGGTTTTGTTCTGCGGCTGTGGGAGTGGGACAACCCCCTCATGTTTTAACAATGGTAGCCAAGCATCCTGCCC

TATATCCAGGTTTCGTACAAAAACATGAGGAAATGGCTTCAGGTCATAACAGATTGCAACCAATTTCTTCGCCGAGATAC

ACAAACTGGGCATGGCAAGTTCATGGAAACAGCAACAATGTCAGCCCAATGCAAGTGTATTCCATTGCAGCATCATCAGG

ATTCCCATCTTCCACAGCTACTACACCTCCATCAGCTACTTACCTTCCTCCGAACCTCCAAGACGGTAGTGCTTCCGCCT

ACAACGTCCGCCGCCTTCCCTTCCCAGCCGCATCCAGCATGTACTAGTGCTTTTGAAAGGCTGGCTAGGTCATGAGCAAA

TGTACTAGTTTGTAATGTGCAGAGATGTTAATTTTTCGTTTCGAATGTGTTTCACATTATCAAACACAGTGGTGTGTATA

TGACCAAACATTGTCCTTGTAACTGTTGTACATGGCCGGCCCAGGCCCAGTTCTGGCAAGGTGACCGTCTAGGGCCCAAA

>MdAP2D64 [Leaf=Malus domestica] AP2 domain class transcription factor

CACAAACAAAACCCAAACAACTCCCTCACACTCAGTCTCTCATCTCCCTCTTTCTCTCTCTCCATTTCTCTCTCTAGGGT

TTTAGTTTGGGTTCAATTGGAGTCTTCAATCATGGCGTCGTCGTCCTCGGATCCCGGTCCGAAGACCGAAAGCGGTGGCG

GCAGTGGCGGTGGGGGTGCAGAGCCGTCGGAGGCGGTGATGGCGAGCGATCAGCTAATGGTGTACAGAGGGGTAAAGAAA

TCGAAGAAAGAGAGAGGATGTACGGCGAAAGAGCGCATCAGCAAGATGCCTCCGTGTGCTGCTGGAAAACGCAGCTCCAT

TTACCGTGGAGTCACTCGGCATAGGTGGACAGGTCGGTATGAGGCTCATCTTTGGGATAAAAGTACTTGGAATCAGAACC

AGAATAAGAAGGGAAAGCAAGTTTACTTGGGGGCCTATGACGATGAAGAGGCTGCAGCTAGAGCTTATGATCTTGCTGCC

TTGAAATATTGGGGTCCTGGCACTCTCATCAATTTTCCTGTTACTGATTACACAAGGGATCTTGAAGAGATGCAGAATCT

GTCAAGGGAGGAATACCTTGCTTCTCTTCGGAGGAAAAGCAGTGGTTTCTCAAGAGGACTTTCTAAGTACCGTGGGCTTT

CCAGTCGTTGGGAGCCGTCACTGGGTCGTATGGGTGGATCTGAGTACTTCAATAGCTTACATTATGGTACAGGCGTTGAT

CCAGCAACAGAAAGTGAACTTTTGGGAGCTTTTTGCATTGAAAGAAAGATTGACCTAACAAGTTACATCAAGTGGTGGGG

ACCCAACAGATCTCGTCAAGCTGAAACTAGCATGAAATCGTCAGAAGAAACAAAACACGGTTGTGTTGGAGATATTGGAG

CTGAACTTAAAACATTGGAATGGGAAATCCAGCCTACTGAACCATACCAGATGCCCCGTTTGGGCATCTCCCATGTAAGT

AAAAAGCATAAAGGTACCAGAGTCTCAGCCATGAGCATCCTTTCAAGGTCAGCGGCATATAAGAACTTGCAAGAGAAAGC

ATCACAAAAGGAGGAAAAAGATGCAGATAATGATGAGAATGAAAACAAAAATACCATCCACAAGATGGACTATGGCAAAG

CAGTTGAGAAATCCACAAGTCATGATGAGGGACTTAGTTCTGCATTAGGAATGAGTGGTGGACCATCTCACCAAAGAGAT

GTGTTCCCATTGGCTCCCTTCTTGTCTGCACCACTTCTGACCAGCTACAGTGCCATCGATCCCTTGGTAGACCCCATTCT

CTGGACGTCTCTTGCTCCTGTTCTTCCTTCTGGAATTTCTCGTCCTACTGAGATTACAAAGACCGAGACTAGTTCAAGTT

TCGCTTTATTTCAGCCCGAAGAATGATGATGCATTTAATTTTGCTCTACTGGTTCCAAAGGGTTCACCCATGCATTACTT

TGCCACAAGAGCAGGAAAAAGGGGCATGAACAGTTGATGTAGCCTTGACAACTGGGCTTTTACTCGTTTCGCAGGTTTGT

GCTATGTAAGGTGTAGAATATATAGAAAAGCAGATAAATTATATTTGAAAACTTTGTTGTCTGCTGATGAGCTGAGATCG

TTGAGCGTGTGTACGTTACTTGTTCTGCATGGTACTTAGTTTTGCTGAATAAGTTCAAGACATGGGTTCTTGATAACATT

TTGTACAGAAAAGATGGTTGTTTATAAAAGTGCACAAATAGAGTCACTGTGCTTTCAAAAAAAA

>MdAP2D65 [Leaf=Malus domestica] AP2 domain class transcription factor

TGAGAGAGAGAGCGAGAGAGATAATGGTAGCATAGATAGATTCAGTCGCAGGAGTTGGGATTTGTTTTAGATGAGTGTTA

GATTTGGTCCTATCAGCTCTGCAGCTACCAAACAAAACACCCAGTTTTGAAAAACCAGAGGCAAAGGGAAGAAGAGAGTG

GGGCAAATAAGGAAGAAGAAAATGGAGAGTATGATTAGTTTATATGTTGCCCAATGATTGAGTTGCCTTGGCACAACAGA

AACTTACCCAACAATCTCTGATCACAAAAATAGAAGGAAGAAATCTCAAAAACCATGAAATCCATGGGTAATGATGGTAA

CAACAGTAACAGTAATAATAACTGGTTGGATTTTTCACTGTCTCCAAACATGAAGATGGAGTCCCCTTCTTCTTCCTCTT

CTGTTGCTGCTAGCTTTTTTCACTCACCACCTCACTTCAGCTATGGAGTTTACTATGGAGTTGAAGAAGGAGGAGGAGGA

GGAGGGGAGGATAACGGTCCAGTCCTGTACTCCCCTCTGTCCATGATGCCACTCAAGTCTGATGGCTCTCTCTGTATTAT

GGAAGCTCTCAACAGGTCCCAGCACCACCCTCACGCTGCAATGGTGACAACAACTAGTCCAACTCCAAAACTAGAGGACT

TCTTTGGAGGTGCAGCCATGGGGACCCATCAATATGAAAGCAGTGACAGAGAAGCCATGGCTCTCAGCTTAGACAGCATG

TTCTACCCCCAAAACCCACACCCAACAACCCAACATCATGTACCCAGCAACCACCAAAACTTGATGAGCCATAGTCTTCA

GCAGCACCAGCAGCAGACCCAGCAACACCAACAACAGTACTCATCATCATACTACTCAGGCCTCAGAACCCATGCGATGA

TCTTGGAGGAGGGTCACAAACAAACCCATGTTTCAGCAGATAATTACAATCTGCACCCAGCAAGAATAGGGCTTGATCAG

AGCATTGCAGAGATGAAGAGTTGGGTTTCCAGAAACAATTCCATGGACCAGAACAACATGGCTGCTGGGTGCATGGGAGA

AAATGGGGGATTGTCCTATGGGGATTTGCAGTGTCTGAGCTTGTCCATGAGCCCTGGCTCACAGTCTAGCTGTGTGACCA

GCTCACAGCAGATTTCTCCCACTGTGACTGCAGATTGTGTGACAATGGTGGTGGATACCAAGAAAAGAGGGCCTGTAAAG

GTGGATCAAAAGCAGATTGTTCACAGGAAGTCATTGGATACATTTGGCCAGAGAACCTCTCAGTACAGAGGAGTCACAAG

GCACCGATGGACCGGTCGATACGAAGCGCATCTGTGGGACAATAGTTGCAAGAAAGAAGGCCAGAGCAGGAAGGGAAGGC

AAGTTTACTTGGGAGGTTATGATATGGAGGAAAAAGCTGCACGAGCTTATGATCTAGCAGCACTTAAGTATTGGGGACCA

TCCACTCACATTAATTTCCCATTGGAAAATTATAAAAAAGAACTAGAGGACATGAAGAATATGACCAGACAAGAATATGT

TGCTCACTTACGAAGAAAAAGCAGTGGATTCTCAAGAGGGGCTTCAATGTATAGAGGAGTTACAAGACATCACCAACATG

GAAGATGGCAAGCTAGGATTGGAAGGGTTGCTGGAAATAAGGATCTTTATCTTGGGACATTCAGCACTCAAGAGGAAGCT

GCTGAGGCTTATGACATAGCTGCAATCAAATTCCGAGGACTGAATGCTGTGACCAACTTTGACATAACACGGTATGATGT

GGACCGAATTATGTCAAGCAATACCCTCCTTGCCGGTGAACTTGCTAAGCGAAATAAGGAGGTTGTACCCATTAATGAGG

CTACTAATGAAACCAATGTTTCGCAAATCGGCAATGGGGAAGCCGTTGTCCCACTGAAGAATATTAGTGAAGGGGAAGAT

TGGAAAATGGCACTCTATCAGTCCTCCCAGCAACTTGATCAGAAGCAACCAAGCACCGAAACTCAGAATGTGATTCAAGC

CCAGGCAGAGGACTCTACCAAAATGGGGAATGCTCATTTTTCCAATGCCTCTTCATTGGTGACAAGCTTAGGCAGCTCAA

GAGAAGCAAGCCCTGATAAGTCAAGCCAGCCTAGTTTCTTCGGAATGCCTCCATCTGCTTCCAAGTTTTTCACAGGTTCG

AGTGATGCAGTGAGTTCTTGGATCCCAACAGTCCAATCCAGGCCAGGACTCACCATTCCTCACATGCCAATTTTCGCTGC

CTGGACAGATGCATAGCCATAGCCATAGGCTTTGTGTTTTTTGCTTATCTGTGGAAAGTTTTTTTCACTAAATTACATGC

GAACGATGGTTCATGTAGTTTTTATCTTAGTGGGGGTAAATTATGGTTAATGATATGGTCTTGGTAGGGCTTTTTGTGAG

CAAAGTAAACGAAAACATCACTGATTTCCCTCATGTTTTAAGCCTATCTTATCAAAATGATGAAAGAAATTGAAGGGAAA

AAGTTTCAAAGA

>MdRAV2 [Leaf=Malus domestica] AP2 domain class transcription factor

GTTTAAAGCACCTTCTTATCTGTCATTCTATCTCTCTGTCTCTCTCTCTCCTCTCCACCTTCGAACACACAACATACATA

ATCAACGAAACAAGAACAATGGATGGAGTAAGTAGCACAGAAGAGAGCACAACCAGTGACTCCATATCCATTTCGCCACC

CCATCATATTGTTACACGTGTGGAGCCACTTGCCAAGTCAGCGCCCCAAGTGGACAGCCTCTGCCGCGTCGGCAGCGGGG

CCAGCAGCGTGATTCTCGACTCCGACCTCAGCAGCGGCGGCGGCACTGGTGGCGTGGAGGCCGAGTCCCGGAAGCTCCCT

TCCTCCAAGTACAAAGGCGTGGTCCCACAGCCCAACGGCCGTTGGGGGGCCCAGATTTACGAGAAGCACCAGCGCGTCTG

GCTGGGCACCTTCAACGAAGAGGACGAGGCCGCTACGGCCTACGACGTTGCTGCACAGCGCTTCCGCGGCCGCGACGCTG

TCACCAACTTTAAGCCCTCCTCTGACGACGAAGAAAACGACGTCGTCGAGGCCGCGTTTCTGAGCTCCCACTCCAAGTCT

GAAATCGTCGACATGCTGCGAAAGCACACGTACAATGACGAACTGGAACAGAGCAAGCGCAACTACTTCTCTTACGGGAA

GCGGGGCCGGTCCAACGGGCCGCTGGGCTTGTTCGGGACGGACAACAGTCGCGTCCAGAAAGCGCGTGAGCAGCTGTTCG

AGAAAGCCGTGACCCCCAGCGATGTCGGGAAGCTGAACCGCCTCGTGATCCCGAAGCAGCACGCCGAAAAGCACTTTCCG

TTGCAAAGCGGAAGCACTGCAACCATAACGGTAAGTGCATCTTCCGCTTGCAAAGGAGTGCTTTTGAATTTTGAGGATGT

CGGGGGAAAAGTGTGGAGGTTTCGATACTCTTACTGGAACAGCAGTCAAAGCTACGTGTTGACCAAAGGATGGAGCCGGT

TCGTGAAGGAGAAGAATCTGAAGGCCGGGGACATTGTGAGCTTTCAGAGGTCAACCGGACCGGACAAACATCTGTATATT

GATTGGAAGACCAGGATGAGTGTGTATAATAACAGTAATGGGTCGAACCCGGTTCAAGGTCAGGTTGCACCGGTTCAGAT

GGTTCGGCTATTTGGGGTCAACATATTCAAAATACCTGGGAGTAGCGGGGCTGGCCCAGTGGATGCTGCAGCTGCCGCTG

CTATTGGCGGCGGTTGCAATAATAATATCGGCAAAAGAATGAGAGAGATGGAGTTTTTGAAATTAGAGTTTAGCAAGAAG

CCTAGGATCATCGGAGCTTTGTAGAATTTTTAAAAATTTTCTTTGTTTTTTTTAGCTTTCTGTGAAGCTGCAAGAAAGGT

GAATTGTATATTAGATGACAAAAAGAATAAAGCTGAGCTGAAGAAAGCAAAGCTTTTGAAATTAGGTGCAACAAATTAAT

TTGAAAAAAAAAAAAA
