# Supplementary material for: Isolation, sequencing, and expression analysis of 30 AP2/ERF transcription factors in apple
Source: PeerJ. 2020 Jan 17;8:e8391. doi: 10.7717/peerj.8391 (PMC6970539; doi:10.7717/peerj.8391)
Supplement: Table S1 [file peerj-08-8391-s004.docx]

Table S1. Application of primers and sequences

| Gene name | Forward Primer Sequence (5'- 3') | Reverse Primer Sequence (5'- 3') |
| --- | --- | --- |
| Complete ORF amplification | |  |
| *MdERF3* | ATGCCGGGGATGAAAACCGTAATC | CTAACTACAAAACTTCATGGGCATA |
| *MdERF4* | ATGGACCCTTCGTTCTTCCAAAAC | TTAGCTCTCGGATATACTGAGTAG |
| *MdERF5* | ATGCTCTCGCCGTCGATTCTTCAT | TCAACTTACGACGTCAGCTAAGAA |
| *MdERF6* | ATGACAACCTTCCAACCCATCTCG | TCAACATACGACGTCAGCCAAGAA |
| *MdERF7* | ATGGTGAAATCGAAAAAGTACAGA | CTAAATTTTTTCCTCCCCTTGATG |
| *MdERF8* | ATGCCTGAGCCTCGGAGACAGCTG | TTATTGGCATACTATGTTGAGGGG |
| *MdERF11* | ATGCCCAAGTTGGTCAAGACCGAT | TCATTCATCATCTCGATCCTCATT |
| *MdERF16* | ATGGAGTCCGAGGCTTCGGACGGG | TCATTTAGGCAGCCCCTGCTCTTC |
| *MdERF17* | ATGGGTTTGCGCGAACAAAACGAC | TTAATTATATGATATGATCCCATC |
| *MdERF18* | ATGGAGGAAAAGTTTCCCAAGATG | TTAGGAATCAGAGACCAGAAGAGC |
| *MdERF19* | ATGTGTGGAGGTGCTATTATTTCC | TCAGAAAGTTCCTCCAACAATTGC |
| *MdERF22* | ATGGTACAATCAAGGAAGTTCAGA | TTAAATATTATTTCCTTCGTGAAC |
| *MdERF23* | ATGGAAACAAGCGTAGATTGGGAG | TCAATCCTGGTCCTTCCAAAAGAG |
| *MdERF24* | ATGGTTTCAGCTCTAGCTCAGGTG | TCATCTTGAGTGACTTCTGTCAAA |
| *MdERF25* | ATGAGAAGGCAACTTGGGGAGGCA | CTAGAAAATCTGACCCTCCATATT |
| *MdERF26* | ATGGCTAGATCCCAGCAGCGGTAT | TTATTCAATAACCATTCATTTAAA |
| *MdERF27* | ATGAAGTCTTTGGATGCGGAGTCG | TCAGAGGACCACCAGCGGGTCGGA |
| *MdERF28* | ATGGCTTTACACTTCTCACAACAA | CTAGATGATCTGACCCTGCATATT |
| *MdERF31* | ATGTCTACTGTAGACGCGCTCCAC | TCAGAACAGCGGCGCCGGTAGATT |
| *MdERF32* | ATGAATTACTCGACTTTTGATTCC | TCACCAATTAGGAGTGGCGCTGGT |
| *MdERF33* | ATGGCTGACCCAACTCACTCGGAG | TTAATGATGCCACAATAAGGCCTC |
| *MdERF34* | ATGGAGGGCGGCGGCGGAGAGGCT | CTAATTCCACTCACCTTCCGAATT |
| *MdERF35* | ATGACAAATTCCGATTCTTTAACC | TCAGGCTCTCTCAGGTGAGTTGGA |
| *MdERF39* | ATGGAACCCCAGGCTGCAGACTGC | CTAGGGATCCCAAATGGACCAATG |
| *MdAP2D60* | ATGAAGTCCATGAATGATCATAAC | CTAGGTCTCATTCCAGGCAGCAAA |
| *MdAP2D62* | ATGGATTCTTCTCCTCAGAACTGG | TTATTCCATCCCAAAAATTGGTGT |
| *MdAP2D63* | ATGTTGGATCTTAACGTTAATTTC | CTAGTACATGCTGGATGCGGCTGG |
| *MdAP2D64* | ATGGCGTCGTCGTCCTCGGATCCC | TCATTCTTCGGGCTGAAATAAAGC |
| *MdAP2D65* | ATGAAATCCATGGGTAATGATGGT | CTATGCATCTGTCCAGGCAGCGAA |
| *MdRAV2* | ATGGATGGAGTAAGTAGCACAGAA | CTACAAAGCTCCGATGATCCTAGG |
| RT-qPCR |  |  |
| *MdERF3* | GGTCAGCAGGATTTCTCTTGG | CGATAACCTCAAACACCCGC |
| *MdERF4* | CGCAGACTATTTGGAGGAGC | CACAGATGGACCAACGTGAT |
| *MdERF5* | GAGTTCGGCGAGTTTAACGT | ATTGCAGCACAGTCCCAAAC |
| *MdERF6* | TCTTGGCTGACGTCGTATGT | GCCACACATCACTTGCAACT |
| *MdERF7* | GCAACATCATCAGAGAGGGC | TCCTCCCCTTGATGAATGCC |
| *MdERF8* | GCTCGATTCGCTTTACCTGG | TCCAACCAGCAAGCAATGTC |
| *MdERF11* | GGCGTTTAGGGCTTATTTGGT | GGCTGGTATCTGAAGAGGGG |
| *MdERF16* | GTGGCATACGGTCCGAGATA | GGTCCTCAGCAGCATCAATG |
| *MdERF17* | TTTATGCTTCCTCTGTGCGG | TGGGGCACGTACTTGAAAGA |
| *MdERF18* | CGTCCGAATCAGCATCATCA | GAGACCCAACTTTGCTCTGC |
| *MdERF19* | CCTTACCCCAGCTTTCCAGT | CACATCCTGCAAGCACAAGT |
| *MdERF22* | CCAACCATCTGAGCTGCTTG | ACAGTGTGGTGGCATTTTATGA |
| *MdERF23* | TCACATCCCACGAAGAACCT | ACGAGTGAGAAATGATGAGCG |
| *MdERF24* | CATCTCAACAAGCCGCAGAG | TCTCCAAGCGTTCACCCTAG |
| *MdERF25* | GGAGGTACAAAATATGGAGGGTC | ACAAACTTTCCCACACACATCT |
| *MdERF26* | CGGAGCAGAACAATGGGTAG | AGCTCCTCAATCATCTGCTCA |
| *MdERF27* | CGACGACGCATTCAGTTTCT | CCGGAACAACTCGCTGAAAT |
| *MdERF28* | GTGGCTGCATCTTCTTCACC | GATTCCGATCTTGGTGTCCC |
| *MdERF31* | TCTGAGATGTTCTGGCCTCC | ATCTTCTTCTCCTCCTGGCC |
| *MdERF32* | GAAAACAAGTACCAGCGCCA | AAAGATTGGAGTCACGGTTCA |
| *MdERF33* | GTTTGCTGATTCGGTGGAGG | CAACTTCAACACACTCGGCA |
| *MdERF34* | GGGAGGTATGTGACTGCTGA | TGGCCAAAATTTCTCCATCCA |
| *MdERF35* | CAGAAGGATGTGCACACCAA | GCGATGATGCTGAGATGTCA |
| *MdERF39* | TGCAGCATTGGTCCATTTGG | GGGCAAAGATACCAAGGCAG |
| *MdAP2D60* | TTGCAAAACCTCCATTGGCA | TAGGTCTCATTCCAGGCAGC |
| *MdAP2D62* | TATGGAAGTTGGATTGCGCC | TCTCACCCTCACATTCACCC |
| *MdAP2D63* | CCACAGCTACTACACCTCCA | TGACCTAGCCAGCCTTTCAA |
| *MdAP2D64* | GCCCGAAGAATGATGATGCA | GCGAAACGAGTAAAAGCCCA |
| *MdAP2D65* | GAATGCCTCCATCTGCTTCC | CTGTCCAGGCAGCGAAAATT |
| *MdRAV2* | AAATACCTGGGAGTAGCGGG | GCTCCGATGATCCTAGGCTT |
| GFP |  |  |
| MdERF28-F | cGagctcGgtaCCCGGggatccATGGCTTTACACTTC | |
| MdERF28-R | ccttgctcaccatGGTgtcgacGATGATCTGACCCTG | |
